# Drug-associated gingival disorders: a retrospective pharmacovigilance assessment using disproportionality analysis

**DOI:** 10.1038/s41405-024-00291-8

**Published:** 2025-03-11

**Authors:** Kannan Sridharan, Gowri Sivaramakrishnan

**Affiliations:** 1 Department of Pharmacology & Therapeutics, College of Medicine & Health Sciences, Arabian Gulf University, Manama, Kingdom of Bahrain; 2 Bahrain Defence Force Royal Medical Services, Riffa, Kingdom of Bahrain

**Keywords:** Bisphosphonates in dentistry, Gum disease

## Abstract

**Background:**

Drug-associated gingival disorders can negatively impact on oral health. This study aimed to utilize the United States Food and Drug Administration Adverse Event Reporting System (USFDA AERS) to comprehensively assess the associations between medications and specific gingival disorders.

**Methods:**

Data were extracted from the USFDA AERS from 2004-2024 using Preferred Terms for eight gingival disorders. Reports were deduplicated and disproportionality analysis was conducted using frequentist and Bayesian approaches to detect potential signals. Volcano plots were generated for each gum disorder to identify the drugs with the strongest signals based on the statistical significance and magnitude of association.

**Results:**

A total of 11,465 reports were included. Several anti-osteoporotic drugs, anti-thrombotics, calcium channel blockers and immunosuppressants showed significant associations with multiple gingival disorders. Phenytoin was linked to hypertrophy and bleeding. Stomatological preparations were associated with discoloration and bleeding. Emergent signals were identified with finasteride, COVID-19 vaccine, and levothyroxine with gum disorders.

**Conclusion:**

This study highlights the need for increased awareness of oral side effects amongst healthcare providers. Future research should explore the mechanisms of drug-induced gingival disorders and develop interdisciplinary management strategies to enhance oral health in patients on long-term medications.

## Introduction

Drug-associated gingival disorders encompass a range of clinical presentations, from gingival hypertrophy and bleeding to discoloration and erosion, and are well-documented. The prevalence of these drug-induced gingival conditions varies significantly depending on both the drug type and the specific disorder, with occurrences ranging from approximately 4% in patients using antihypertensives to as high as 70% in prescribed anticonvulsants, particularly in cases of gingival hypertrophy [1]. Although locally applied drugs have long been implicated, systemically administered medications can also profoundly impact the inflammatory and immune responses within periodontal tissues, particularly the gingival area [2].

Emerging studies reveal elevated levels of specific biomarkers associated with gingival fibrosis in patients with drug-induced gingival disorders. Particularly, increased expression of connective tissue growth factor and markers indicative of epithelial-to-mesenchymal transition have been detected in gingival lesions linked to anticonvulsants, calcium channel blockers, and immunosuppressants. Recently, additional fibrosis-related markers, such as periostin and members of the lysyl oxidase enzyme family, have been documented, indicating a complex pathophysiology underlying these drug-induced effects [3]. Gingival hypertrophy is particularly prevalent among users of immunosuppressants, anticonvulsants, and calcium channel blockers [4], while other medications, including beta-blockers and oral contraceptives, are more commonly associated with gingivitis [5]. Additionally, oral contraceptives and anticonvulsants have been implicated in cases of gingival discoloration, underscoring the range of periodontal side effects that can accompany drug therapy [6].

The clinical impact of gingival enlargement extends beyond physical symptoms; this condition can present as a cosmetic concern, which may contribute to decreased medication compliance among affected patients [7]. Of all the drug-associated gingival disorders, gingival hypertrophy has been the most extensively studied. The condition often manifests within two to four months following drug initiation, usually without accompanying pain. Initial signs typically present as bead-like swellings of the interdental papillae, which may progressively involve the marginal gingiva. In cases lacking secondary inflammation, the enlargement has a distinctive firm, pink, mulberry-like appearance with small lobulations and minimal bleeding upon probing [8]. This condition is most prominent in the anterior maxillary and mandibular regions but does not develop in edentulous areas, and the affected gingiva may regress in regions where teeth have been extracted. However, if secondary inflammation supervenes, the existing hypertrophy may increase in size, taking on the characteristics of inflammatory swelling.

The United States Food and Drug Administration Adverse Event Reporting System (USFDA AERS) is a vital pharmacovigilance resource, providing publicly accessible adverse event reports from manufacturers (mandatory reporting) and healthcare providers (voluntary reporting) through a spontaneous reporting system [9]. Disproportionality analysis, a cornerstone methodology in pharmacovigilance, utilizes various statistical techniques to detect potential safety signals by comparing event frequencies across different drugs within adverse event databases [10]. To date, only one retrospective pharmacovigilance analysis in the USFDA AERS database has specifically examined drug-associated gingival hyperplasia, identifying signals with several immunosuppressants, anti-epileptics, and calcium channel blockers [11]. Another study utilizing the French pharmacovigilance database focused on drug-induced gingival overgrowth; however, it primarily provided descriptive findings without employing any signal detection methods [12].

Despite these insights, there remains a gap in the literature on the broader spectrum of drug-associated gingival disorders, including but not limited to gingival atrophy, bleeding, discoloration, erosion, hyperpigmentation, hypoplasia, and gingivitis. The specific gingival disorders included in the study were selected because they are among the most observed conditions, ensuring clinical relevance and broader applicability of the findings. Recognizing this unmet need, we undertook the present study to provide a comprehensive pharmacovigilance assessment of these disorders. Our goal is to elucidate the full scope of gingival adverse events associated with various medications, leveraging real-world data to inform clinicians and improve patient care in drug-induced periodontal management.

## Methods

### Data source

Data for this disproportionality study was obtained from the USFDA AERS, using the Standardized Medical Dictionary for Regulatory Activities (MedDRA version 27.1) Preferred Terms (PTs) [13]. The following PTs were individually queried to capture reports: gingival atrophy (MedDRA PT code: 10018275), gingival bleeding (MedDRA PT code: 10018276), gingival discoloration (MedDRA PT code: 10018278), gingival erosion (MedDRA PT code: 10018282), gingival hyperpigmentation (MedDRA PT code: 10049580), gingival hypertrophy (MedDRA PT code: 10018284), gingival hypoplasia (MedDRA PT code: 10018285), and gingivitis (MedDRA PT code: 10018292). These gingival disorders were selected due to their common occurrence in clinical practice, and the PTs were chosen to enable separate analyses of demographic data and disproportionality measures for each specific gum disorder. Data was collected from adverse event reports submitted over 82 quarters, spanning from March 2004 to June 2024.

### Data processing

We queried the USFDA AERS database for each PT independently, ensuring comprehensive retrieval of individual case safety reports without restrictions [14]. Following the USFDA’s deduplication guidelines, reports were sorted in ascending order by Case_ID, retaining the most recent submission (determined by the highest FDA_DT or Individual Safety Report number) to eliminate duplicate records. Only reports in which the suspected drug had a “primary suspect” role were included in the analysis, and we considered only non-proprietary drug names. The deduplication process was performed separately for each PT. Demographic details such as age, gender, report year, and reporting country were extracted for each adverse event of interest. Missing data for any variable were categorized as ‘not specified’.

### Data mining algorithms

For signal detection, we used a “case-non-case” disproportionality analysis approach, which compares the frequency of exposure to the drug of interest in cases (specific gum disorders) versus non-cases (all other reported events) [15]. The OpenVigil 2.1 platform was used to retrieve data on drug-gum disorder pairs. Four data mining algorithms, two frequentists and two Bayesian, were employed to detect potential signals. The Council for International Organizations of Medical Sciences (CIOMS) Working Group VIII report emphasizes that there is no single “gold standard” for signal detection. Instead, it recommends using a combination of statistical approaches from both frequentist and Bayesian paradigms for more robust signal identification [16].

Frequentist approaches included the Reporting Odds Ratio (ROR) and the Proportional Reporting Ratio (PRR). The ROR is estimated comparing the odds of specific gum disorder being reported for the drug of interest to the odds of the same event being reported for all other drugs in the database. The PRR is estimated as the ratio of proportion of specific gum disorder being reported with the drug of interest to the proportion of the same adverse event reported for all other drugs. Signal detection using frequentist methods adhered to Evan’s criteria, which require a minimum of three reports, a PRR of 2 or greater, and a chi-square (χ²) value of 4 or more per drug-gum disorder pair [17]. ROR was calculated with a 95% confidence interval (CI), and a signal was deemed significant if the lower CI limit exceeded one. PRR, analogous to relative risk, provided insight into the relative occurrence of events for the drug of interest compared to other drugs.

Bayesian methods included the Bayesian Confidence Propagation Neural Network (BCPNN) and the Multi-Item Gamma Poisson Shrinker (MGPS). The Information Component (IC) from BCPNN was employed as a measure for signal detection, defined as the logarithmic ratio of the joint probability of the drug-gum disorder pair to the product of their individual probabilities in the database. An IC-based signal was present when the lower bound of the IC’s 95% CI (IC025) exceeded zero. In the MGPS method, the Empirical Bayes Geometric Mean (EBGM) was used, with signal detection criteria met if the lower 95% CI bound (EBGM05) exceeded 2. We considered only generic drug names, and drugs were categorized using the Anatomical Therapeutic Chemical (ATC) classification system at the first hierarchical level, as specified by the World Health Organization [18].

This study complies with the Reporting of a Disproportionality analysis for drUg Safety signal detection using spontaneously reported adverse events in Pharmacovigilance (READUS-PV) guidelines [19].

### Statistical analysis

Descriptive statistics were used to summarize demographic variables, with numerical variables presented as means (SD) and categorical variables as proportions (%). Volcano plots were generated for each gum disorder, with log_2_(ROR) plotted on the X-axis and -log_10_(*P*-values) on the Y-axis, highlighting the top 10 drugs based on fold change and statistical significance thresholds. All statistical analyses were conducted using SPSS (IBM Corp. Released 2020. IBM SPSS Statistics for Windows, Version 27.0. Armonk, NY: IBM Corp.), and VolcaNoseR was used for generating volcano plots [20].

## Results

### Search results

**Fig. 1:**
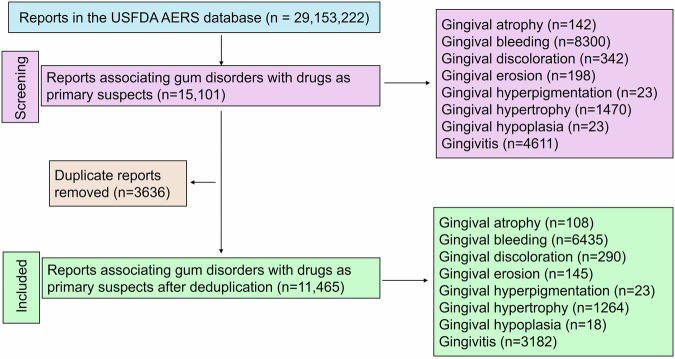
Study flow diagram. A total of 11,465 unique reports were included in the final analysis for drug-associated gum disorders.

**Fig. 2:**
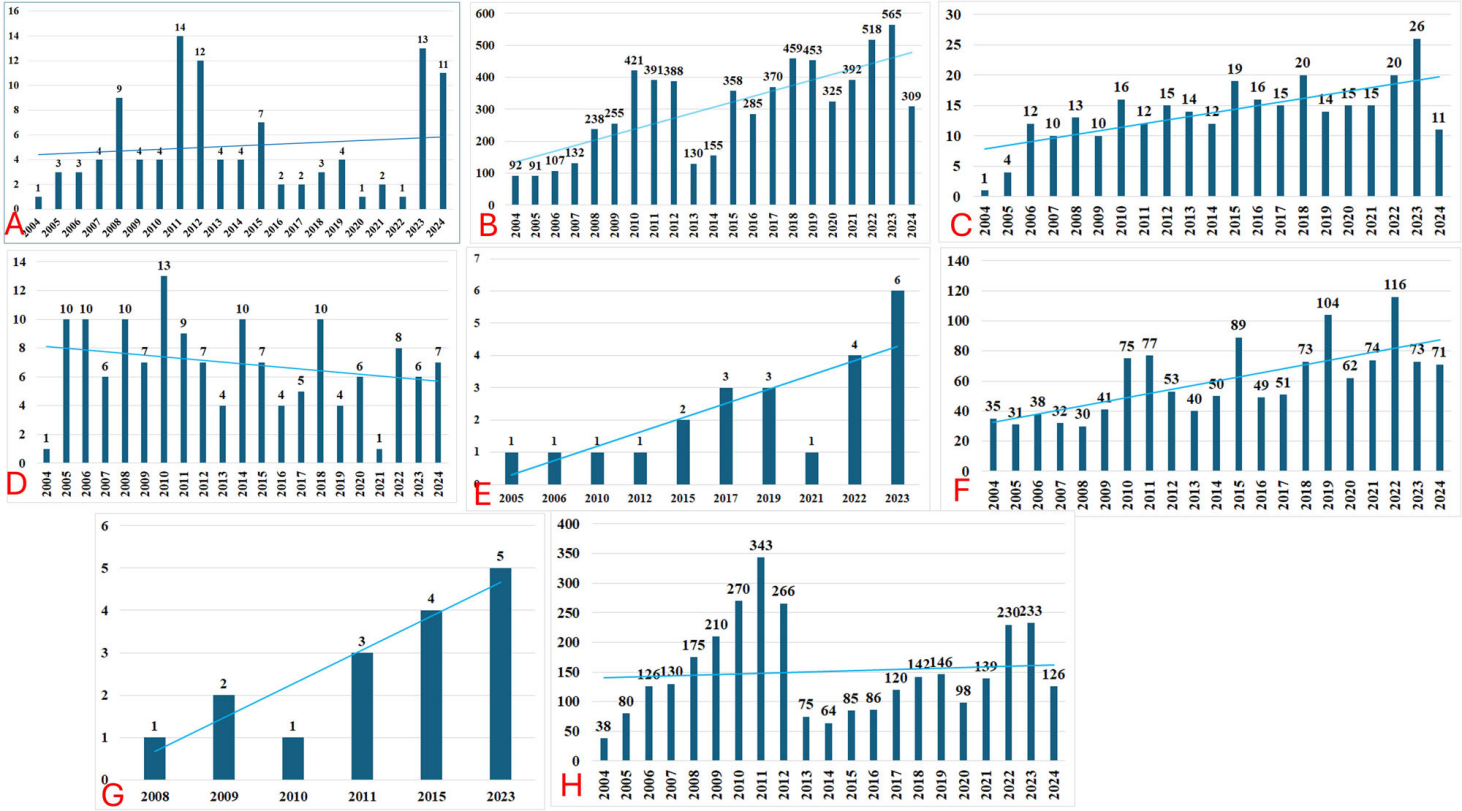
Trend in timeline of reporting of gum disorders in the USFDA AERS database. **A** Gingival atrophy; **B** Gingival bleeding; **C** Gingival discoloration; **D** Gingival erosion; **E** Gingival hyperpigmentation; **F** Gingival hypertrophy; **G** Gingival hypoplasia; and **H** Gingivitis. The blue lines across the bars represent the trendline for reporting years.

A total of 29, 163, 222 reports exist in the USFDA AERS database of which finally 11, 465 were included for the analysis (Fig. 1). Summaries of demographic characteristics of study participants for various gum disorders are outlined in Table 1. Most were in the middle-age group (≥40 to <65 years) and were females. Time trend analysis revealed increased reporting rates for all drug-associated gingival disorders except gingival erosion (Fig. 2).

**Table 1:** Summary of demographic characteristics for drug-associated gum disorders.

Characteristics		Gingival atrophy (*n* = 108)	Gingival bleeding (*n* = 6435)	Gingival discoloration (*n* = 290)	Gingival erosion (*n* = 145)	Gingival hyperpigmentation (*n* = 23)	Gingival hypertrophy (*n* = 1264)	Gingival hypoplasia (*n* = 18)	Gingivitis (*n* = 3182)
Age groups [*n* (%)]	<18	2 (2)	179 (2.8)	7 (3)	4 (3)	0	180 (15)	0	43 (2)
	≥18 to <40	9 (8)	604 (9.4)	41 (14)	21 (15)	3 (13)	155 (12)	1 (6)	237 (7)
	≥40 to <65	23 (21)	2007 (31.2)	81 (28)	42 (29)	10 (44)	369 (29)	6 (33)	1016 (32)
	≥65	20 (18)	1590 (24.7)	47 (16)	33 (23)	5 (22)	201 (16)	2 (11)	854 (27)
	Not specified	39 (36)	2055 (31.9)	114 (39)	45 (31)	5 (22)	359 (28)	10 (56)	1032 (32)
Quantitative age (years)	Mean (SD)	56.5 (17.41)	56.1 (18.5)	50.8 (17.7)	54.41 (18.3)	58.16 (18.0)	44.4 (22.5)	48.6 (13.04)	58.13 (15.9)
	Median (range)	60 (0–85)	59 (0–96)	53 (0–85)	59 (6–91)	60.5 (21–80)	50 (0–87)	46 (27–67)	61 (0–96)
Gender [*n* (%)]	Male	30 (28)	2062 (32)	74 (26)	45 (31)	6 (26)	550 (44)	10 (55)	774 (54)
	Female	72 (67)	3832 (59.5)	192 (66)	89 (62)	16 (70)	557 (44)	3 (17)	2231 (70)
	Unknown	6 (5)	541 (8.4)	24 (9)	11 (8)	1 (4)	157 (13)	5 (28)	177 (6)
Reporting year [*n* (%)]	2004–2008	20 (19)	660 (10.3)	40 (14)	37 (25)	2 (8)	166 (13)	1 (6)	549 (17)
	2009–2012	34 (31)	1456 (22.6)	53 (18)	36 (25)	2 (8)	246 (20)	6 (33)	1089 (34)
	2013–2016	17 (16)	928 (14.4)	61 (21)	25 (17)	5 (20)	228 (18)	4 (22)	310 (10)
	2017–2020	10 (9)	1607 (25)	64 (22)	25 (17)	6 (25)	290 (23)	0	506 (16)
	2021–2024 (June)	28 (26)	1784 (27.7)	72 (25)	22 (15)	11 (45)	334 (27)	7 (39)	728 (23)
Reporting top countries		China; Canada; Denmark; Germany; Japan; UK; USA	UK; USA; NL; France	Brazil; Canada; Japan; India; Germany; USA; Korea; UAE	Australia; UK; USA; India; Japan	USA; UK; Brazil; Greece; Japan	USA; NL; UK; Australia	UK; USA; NZ; France	Australia; Austria; France; USA; UK; Italy; NZ; Canada

*UK* The United Kingdom, *USA* The United States of America, *UAE* The United Arab Emirates, *NL* Netherlands, *NZ* New Zealand.

### Signal detection measures for drug-associated gum disorders

#### Gingival atrophy and gingival discoloration

Signal detection results for gingival atrophy and gingival discoloration are presented in Table 2. Both frequentist and Bayesian measures indicated significant associations for several drugs. For gingival atrophy, signals were observed with clindamycin, oxaliplatin, cortisone, lansoprazole, fluticasone, salmeterol, anti-osteoporotic drugs (denosumab, alendronic acid, and zoledronate), anastrozole, ocrelizumab, haloperidol, conjugated estrogens, finasteride, and COVID-19 vaccines.

**Table 2:** Signal detection measures for drug-associated gingival atrophy and discoloration.

ATC	Drugs	PRR	χ2	RRR	ROR	Lower limit of 95% CI of ROR	Upper limit of 95% CI of ROR	IC025	EBGM05	Number of cases
Gingival atrophy										
Analgesics	Buprenorphine	4.1	4.2	4.0	4.1	1.3	12.9	0.6	1.3	3
Antibacterials for systemic use	Clindamycin	22.0	58.2	21.2	22.0	8.1	59.7	1.6	7.8	4
Antineoplastic agents	Oxaliplatin	8.4	12.6	8.2	8.4	2.7	26.6	1	2.6	3
Calcium channel blockers	Amlodipine	4.1	12.3	3.9	4.1	1.9	8.7	0.9	1.8	7
Corticosteroids for systemic use	Cortisone	74.8	213.7	72.0	74.8	27.5	203.1	2.3	26.5	4
Drugs for acid related disorders	Lansoprazole	7.8	27.6	7.4	7.8	3.4	17.8	1.3	3.3	6
Drugs for obstructive airway diseases	Fluticasone	6.3	16.4	6.0	6.3	2.6	15.4	1.1	2.5	5
	Salmeterol	8.4	24.3	8.0	8.4	3.4	20.5	1.2	3.3	5
Drugs for treatment of bone diseases	Denosumab	7.2	34.1	6.8	7.2	3.5	14.8	1.3	3.3	8
	Alendronic acid	27.2	141.4	25.5	27.2	12.6	58.5	2.2	11.8	7
	Zoledronate	12.7	31.0	12.3	12.7	4.7	34.5	1.3	4.5	4
Endocrine therapy	Anastrozole	18.6	33.0	18.1	18.6	5.9	58.7	1.3	5.8	3
Immunostimulants	Interferon beta-1a	3.3	4.3	3.3	3.3	1.2	9.1	0.6	1.2	4
Immunosuppressants	Ocrelizumab	18.3	62.4	17.5	18.3	7.5	45.0	1.7	7.1	5
Psychoanaleptics	Escitalopram	5.1	6.1	5.0	5.1	1.6	16.2	0.7	1.6	3
Psycholeptics	Haloperidol	14.9	25.5	14.5	14.9	4.7	46.9	1.2	4.6	3
Sex hormones and modulators of the genital system	Conjugated estrogens	17.7	88.0	16.6	17.7	8.2	38.0	1.9	7.7	7
Urologicals	Finasteride	101.3	1406.6	84.6	101.3	61.1	168.1	3.9	51	18
Vaccines	Moderna covid-19 vaccine	415.9	837.5	404.4	417.3	132.2	1317.5	2.7	128.1	3
Vitamins	Cholecalciferol	3.6	5.0	3.5	3.6	1.3	9.8	0.7	1.3	4
Not classified	Leuprolide	6.3	8.4	6.2	6.3	2.0	19.9	0.8	2	3
	Tozinameran	236.7	474.6	230.2	237.2	75.2	748.1	2.5	73	3
**Gingival discoloration**										
Antibacterials for systemic use	Metronidazole	5.6	10.7	5.5	5.6	2.1	15.0	0.9	2.1	4
	Linezolid	8.6	13.1	8.5	8.6	2.8	26.8	1	2.7	3
	Moxifloxacin	6.0	7.9	6.0	6.0	1.9	18.8	0.8	1.9	3
Antiepileptics	Lamotrigine	3.9	12.1	3.8	3.9	1.9	8.3	0.9	1.8	7
Antiepileptics	Phenytoin	6.7	13.9	6.6	6.7	2.5	18.0	1	2.5	4
Antihypertensives	Clonidine	4.5	5.0	4.5	4.5	1.5	14.1	0.7	1.4	3
Anti-inflammatory and antirheumatic products	Naproxen	4.6	21.0	4.5	4.6	2.4	8.9	1.1	2.3	9
Antineoplastic agents	Niraparib	13.5	45.1	13.2	13.5	5.6	32.6	1.5	5.5	5
	Nilotinib	6.1	8.2	6.1	6.1	2.0	19.1	0.8	1.9	3
Drugs for acid related disorders	Dexlansoprazole	6.6	13.7	6.5	6.6	2.5	17.8	1	2.4	4
Drugs for obstructive airway diseases	Salmeterol	3.0	4.8	3.0	3.0	1.2	7.3	0.7	1.2	5
	Tiotropium	2.9	4.5	2.9	2.9	1.2	7.1	0.6	1.2	5
Drugs for treatment of bone diseases	Denosumab	4.2	27.6	4.1	4.2	2.4	7.4	1.2	2.3	13
	Zoledronic acid	6.3	25.2	6.1	6.3	3.0	13.2	1.2	2.9	7
	Alendronic acid	5.5	10.4	5.4	5.5	2.0	14.7	0.9	2	4
	Ibandronate	6.7	9.2	6.6	6.7	2.1	20.8	0.9	2.1	3
	Pamidronic acid	45.2	88.2	44.7	45.2	14.5	141.1	1.8	14.3	3
Immunosuppressants	Lenalidomide	2.3	7.0	2.2	2.3	1.3	4.1	0.6	1.2	12
Muscle relaxants	Methocarbamol	16.2	28.7	16.1	16.2	5.2	50.7	1.2	5.2	3
Other nervous system drugs	Nicotine	13.8	157.4	13.2	13.8	8.2	23.3	2.2	7.8	15
	Varenicline	3.1	5.1	3.1	3.1	1.3	7.5	0.7	1.3	5
Psychoanaleptics	Bupropion	3.7	10.8	3.6	3.7	1.7	7.8	0.9	1.7	7
	Duloxetine	2.8	5.1	2.7	2.8	1.2	6.2	0.6	1.2	6
Psycholeptics	Zopiclone	7.7	16.8	7.6	7.7	2.9	20.6	1.1	2.8	4
Stomatological preparations	Minocycline	88.6	1305.4	83.5	88.8	54.4	145.0	3.9	51.1	17
	Chlorhexidine gluconate	45.7	89.3	45.2	45.7	14.7	142.7	1.7	14.9	3
	Hydrogen peroxide	358.9	735.1	355.2	361.9	115.5	1133.8	2.7	113.4	3
	Sodium fluoride	64.4	127.9	63.8	64.5	20.7	201.4	1.9	20.4	3
Vitamins	Ergocalciferol	6.3	17.1	6.2	6.3	2.6	15.3	1.1	2.6	5
	Vitamin B 12	4.9	5.8	4.9	4.9	1.6	15.4	0.7	1.6	3

*ATC* Anatomical Therapeutic Chemical, *PRR* Proportional reporting ratio, *χ2* Chi square, *RRR* Relative reporting ratio, *ROR* Reporting odds ratio, *IC* Information component, *EBGM* Empirical Bayes geometric mean.

For gingival discoloration, drugs with significant signals included systemic antibacterials (metronidazole and linezolid), phenytoin, naproxen, niraparib, dexlansoprazole, anti-osteoporotic drugs (denosumab, zoledronic acid, ibandronate, and pamidronic acid), methocarbamol, nicotine, zopiclone, stomatological preparations (minocycline, chlorhexidine, hydrogen peroxide, and fluoride), and ergocalciferol.

### Gingival bleeding

Supplementary Table 1 details the signal detection measures for drugs associated with gingival bleeding. Both frequentist and Bayesian methods detected significant associations with a wide range of drugs, including candesartan, loperamide, phenytoin, primidone, amorolfine, allopurinol, various antihemorrhagics (e.g., eltrombopag, coagulation factors VIII and IX, emicizumab, romiplostim, and tranexamic acid), doxazosin, glucosamine, mefenamic acid, isoniazid, numerous antineoplastic agents (e.g., bevacizumab, niraparib, cabozantinib, sunitinib, sorafenib), and anti-thrombotic drugs (e.g., heparin, rivaroxaban, apixaban, warfarin, clopidogrel, and dabigatran). Additional drugs with signals included ribavirin, telaprevir, sotalol, amlodipine, and a range of anti-osteoporotic drugs.

### Gingival erosion, hyperpigmentation, and erosion

Table 3 summarizes the signal detection results for gingival erosion, hyperpigmentation, and hypoplasia. Gingival erosion showed significant signals with naloxone, anti-osteoporotic drugs (zoledronic acid, pamidronic acid, denosumab, and alendronic acid), thalidomide, statins (atorvastatin and simvastatin), nicotine, and sodium monofluorophosphate. For gingival hyperpigmentation, minocycline, hydroxychloroquine, and methotrexate generated positive signals, while amlodipine, indapamide, and ramipril were associated with gingival hypoplasia.

**Table 3:** Signal detection measures for drug-associated gingival erosion, hyperpigmentation and hypoplasia.

ATC	Drug	PRR	χ2	RRR	ROR	Lower limit 95% CI of ROR	Upper limit 95% CI of ROR	IC025	EBGM05	Number of reports
Gingival erosion										
Analgesics	Buprenorphine	4.1	6.3	4.0	4.1	1.5	11.0	0.7	1.5	4
Anesthetics	Fentanyl	3.9	5.7	3.8	3.9	1.4	10.4	0.7	1.4	4
Antibacterials for systemic use	Sulfamethoxazole	4.3	4.6	4.3	4.4	1.4	13.6	0.7	1.4	3
	Trimethoprim	4.0	4.0	4.0	4.0	1.3	12.6	0.6	1.3	3
Antiepileptics	Lamotrigine	4.5	7.4	4.4	4.5	1.7	12.1	0.8	1.6	4
	Valproic acid	5.4	6.7	5.3	5.4	1.7	17.0	0.8	1.7	3
Antineoplastic agents	Capecitabine	4.2	4.4	4.2	4.2	1.3	13.2	0.7	1.3	3
Drugs for constipation	Naloxone	9.3	14.4	9.1	9.3	3.0	29.2	1.0	2.9	3
Drugs for treating bone diseases	Zoledronic acid	47.7	845.5	40.3	47.7	30.6	74.5	3.4	25.9	23
	Pamidronic acid	285.9	2136.9	268.2	286.8	145.9	563.6	4.1	136.5	9
	Denosumab	4.6	15.3	4.4	4.6	2.1	9.8	1.0	2.1	7
	Alendronic acid	11.1	26.7	10.8	11.1	4.1	30.1	1.3	4.0	4
Immunosuppressants	Thalidomide	10.9	17.5	10.7	10.9	3.5	34.1	1.1	3.4	3
Lipid modifying agents	Atorvastatin	4.7	23.8	4.5	4.7	2.5	9.0	1.1	2.3	10
	Simvastatin	6.3	32.5	5.9	6.3	3.2	12.3	1.3	3.0	9
Other nervous system drugs	Nicotine	11.0	42.9	10.5	11.0	4.8	24.8	1.5	4.7	6
Psychoanaleptics	Duloxetine	3.7	5.3	3.7	3.7	1.4	10.1	0.7	1.4	4
Stomatological preparations	Sodium monofluorophosphate	1443.0	2939.2	1413.2	1466.8	463.2	4644.8	3.3	446.3	3
Thyroid therapy	Levothyroxine	3.0	8.4	2.9	3.0	1.5	6.2	0.8	1.4	8
Vitamins	Vitamin D	5.3	9.8	5.2	5.3	2.0	14.4	0.9	1.9	4
Gingival hyperpigmentation										
Antibacterials for systemic use	Minocycline	759.2	3471.1	495.5	759.8	322.1	1792.7	3.8	210.0	8
Antiprotozoals	Hydroxychloroquine	49.6	150.0	39.0	49.6	18.4	133.6	2.0	14.5	5
Antineoplastic agents	Methotrexate	12.7	33.2	10.1	12.7	4.7	34.1	1.2	3.8	5
Gingival hypoplasia										
Calcium channel blockers	Amlodipine	91.8	349.2	36.3	91.8	35.6	236.9	2.0	14.1	11
Diuretics	Indapamide	318.9	753.9	248.2	319.0	105.0	969.2	2.6	81.7	4
Agents acting on the renin-angiotensin system	Ramipril	52.0	118.3	40.7	52.0	17.1	158.0	1.8	13.4	4

*ATC* Anatomical Therapeutic Chemical, *PRR* Proportional reporting ratio, *χ2* Chi square, *RRR* Relative reporting ratio, *ROR* Reporting odds ratio, *IC* Information component, *EBGM* Empirical Bayes geometric mean.

### Gingival hypertrophy

Drugs associated with gingival hypertrophy are listed in Supplementary Table 2. Significant signals were found with drugs impacting the renin-angiotensin system (e.g., ramipril, candesartan, benazepril), anti-epileptics (e.g., phenytoin, valproic acid, levetiracetam), and calcium channel blockers (e.g., amlodipine, nifedipine, diltiazem). Other drugs with signals included immunosuppressants (e.g., cyclosporine, mycophenolate mofetil), anti-diabetic agents (e.g., gliclazide, glimepiride), beta-blockers (e.g., atenolol, propranolol), and various other classes.

### Gingivitis

Supplementary Table 3 provides signal detection measures for gingivitis-associated drugs. Key drugs with significant signals included candesartan, naratriptan, prilocaine, erythropoietin, selective serotonin receptor antagonists (e.g., granisetron, palonosetron), anti-epileptics (e.g., phenytoin, cannabidiol), corticosteroids (e.g., dexamethasone, cortisone), and a wide range of antineoplastic drugs (including docetaxel, lenvatinib, vinorelbine). Additional signals were found for anti-osteoporotic drugs, stomatological preparations (chlorhexidine, fluoride), COVID-19 vaccines, and ergocalciferol.

### Top 10 drugs associated with gum disorders based on volcano plots

**Fig. 3:**
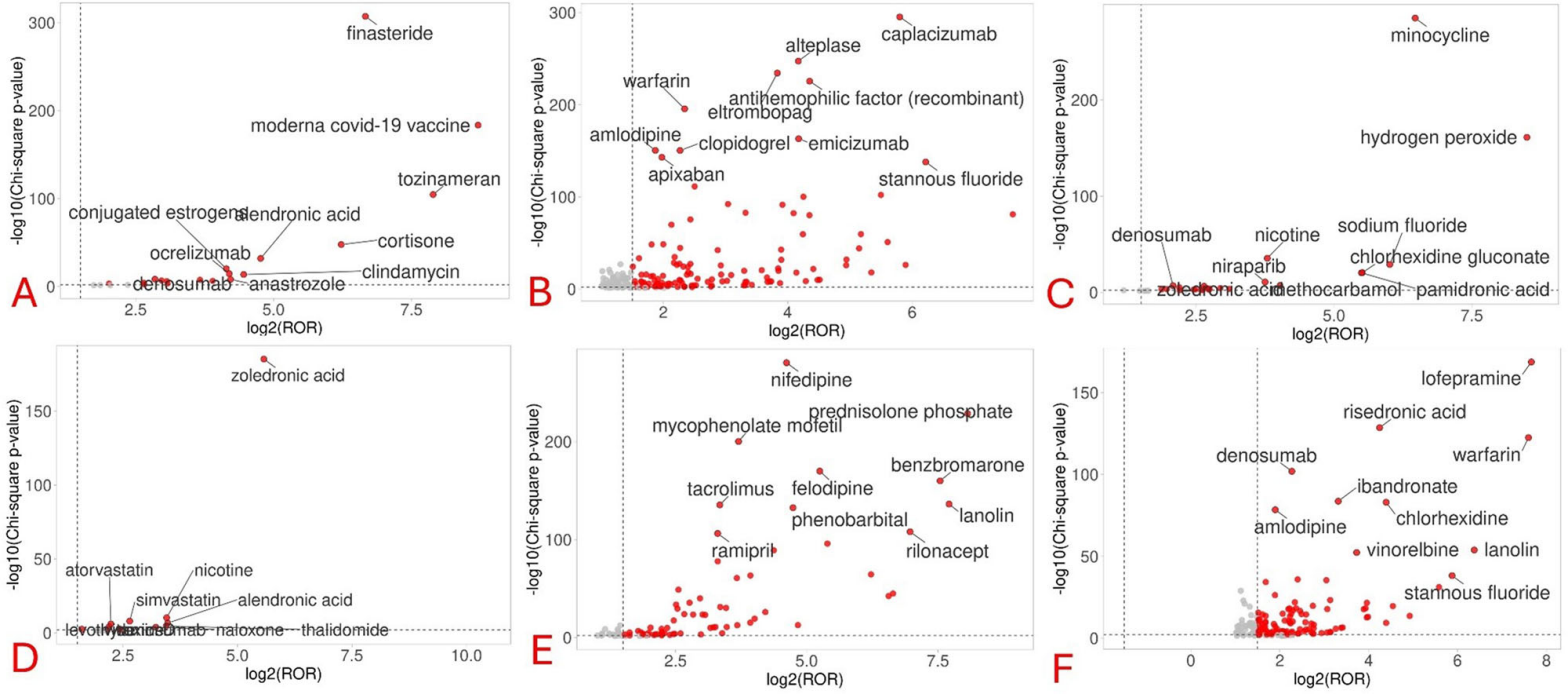
Volcano plots for identifying top 10 drugs for each gum disorder. **A** Gingival atrophy; **B** Gingival bleeding; **C** Gingival discoloration; **D** Gingival erosion; **E** Gingival hypertrophy; and **F** Gingivitis. The red circles represent the location of drugs based on their statistical associations and reporting odds ratios. As far they lie on both the x- and y-axes, more significant is the strength of association of the drug with the concerned gum disorder.

Figure 3 depicts the volcano plots for identifying top drugs associated with each gum disorder. Anti-osteoporotic drugs were the most common top drugs associated with most of the gum disorders except gingival bleeding and hypertrophy. Anti-thrombotics were the key drugs associated with gingival bleeding. Volcano plots could not be generated for gingival hyperpigmentation and hypoplasia due to signals associated with a few numbers of drugs.

## Discussion

### Key findings

The key findings of this study reveal significant associations between various drugs and specific gum disorders, underscoring the potential oral side effects of commonly used medications. Anti-osteoporotic drugs (e.g., denosumab, zoledronic acid, alendronic acid) were consistently linked to multiple gum disorders, including gingival atrophy, discoloration, erosion, and hypertrophy, suggesting a broad spectrum of gingival risks with this class. Remarkably, anti-thrombotic agents (e.g., rivaroxaban, apixaban, warfarin) were predominantly associated with gingival bleeding, while calcium channel blockers and immunosuppressants showed strong signals for gingival hypertrophy. Additionally, phenytoin, a well-known culprit in gingival disorders, was implicated in both gingival hypertrophy and bleeding, reinforcing its adverse oral effects profile. Stomatological preparations such as fluoride and chlorhexidine were also linked to gingival discoloration and bleeding.

### Comparison with existing literature

The results of our study underscore the critical associations between various pharmacological agents and the prevalence of gum disorders, highlighting a need for heightened awareness in both clinical practice and pharmacovigilance. Our findings indicate that anti-osteoporotic drugs, particularly bisphosphonates and denosumab, are significantly linked to multiple gum disorders, including atrophy, discoloration, erosion, hyperpigmentation, and gingivitis. These results are consistent with recent investigations related to the use of bisphosphonates and denosumab in utilizing the USFDA AERS database, where pamidronate was identified as having the highest statistical measures of association with oral adverse effects, while denosumab demonstrated weaker associations [21]. The detrimental effects of bisphosphonates on oral cell types, including gingival fibroblasts, oral keratinocytes, and periodontal ligament cells are well-documented in the literature. These drugs have been shown to impede cell proliferation, migration, and metabolism while increasing apoptosis, ultimately compromising periodontal health [22, 23]. This raises an important consideration for clinicians prescribing these medications, as they may inadvertently contribute to the deterioration of gingival tissues. Additionally, our study identified conjugated estrogens as a significant factor associated with gingival atrophy. The influence of estrogen on vascular dynamics, along with progesterone’s role in promoting inflammatory mediators, can lead to enhanced gingival edema and inflammation. It is noteworthy that estrogen has been shown to exert a dose-dependent reduction in collagen synthesis in human periodontal ligament cells, significantly decreasing pro-collagen I production by over 40% at physiological concentrations in young adult women [24–26]. This suggests that hormonal treatments, particularly in women, may warrant closer monitoring for oral side effects, especially as a recent study found a substantial prevalence of gingival diseases among women using oral contraceptives, with many receiving inadequate dental care [27]. Despite the documented associations with bisphosphonates, denosumab, and hormonal therapies, there remains a striking gap in clinical guidelines, as neither package inserts nor standard recommendations advocate for routine dental monitoring of patients on these medications [28–31]. This oversight highlights a critical need for interdisciplinary communication between healthcare providers and dental professionals to ensure comprehensive patient care.

Emergent signals in our study, such as the association of finasteride with gum atrophy, are particularly intriguing. Finasteride, by inhibiting the conversion of testosterone to dihydrotestosterone, a hormone known to promote fibroblast activity during gingival inflammation—may disrupt normal gingival homeostasis [32]. Notably, research has established a statistically significant positive correlation between testosterone levels and gingival enlargement in young patients undergoing orthodontic therapy [33]. This warrants further investigation into the hormonal influences on periodontal health, particularly in patients receiving finasteride therapy.

Furthermore, we observed an association between gingival discoloration and minocycline, a finding consistent with existing literature that attributes the drug’s pro-anabolic effects on connective tissue to its therapeutic role. However, this effect appears to be mitigated by finasteride, suggesting complex interactions that require further study [34]. The identification of COVID-19 vaccines as a potential contributor to gum atrophy is particularly noteworthy. While reports have surfaced regarding oral adverse effects post-vaccination, such as gingival bleeding and oral ulcers, our study indicates a broader spectrum of possible connections that merit additional research. Klugar et al. [35] documented a range of oral adverse effects such as vesicles being the most common (6.3%). Other reported effects included gingival bleeding (4.3%), halitosis (3.7%), oral paresthesia (2.2%), swollen mucosa (2.2%), and ulcers (2%), with symptoms appearing 1–28 days post-vaccination in healthcare workers following vaccination, further emphasizing the need for vigilance in monitoring oral health post-vaccination [35]. A case series on painful oral lesions following COVID-19 vaccinations revealed occurrences of swelling and pain in the posterior palatal area, central incisor region, mucosa of the lip and lower gingiva, right preauricular area, buccal mucosa, tongue, and the right lower molar region, with some cases showing ulceration and oral mucosal swelling, that were generally mild and resolved quickly with medication [36]. Although a study indicated that COVID-19 patients with gum disease were 3.5 times more likely to be admitted to intensive care, 4.5 times more likely to require a ventilator, and nearly nine times more likely to die than those without gum disease [37], the relationship between COVID-19 vaccine and gum disorders are not well elucidated.

Moreover, methocarbamol was associated with gingival discoloration, an effect likely linked to its phenolic metabolites, which are known to cause urinary discoloration [38]. The relationship between levothyroxine and gingival erosion identified in our study is also significant, as thyroid hormones play a crucial role in bone remodeling and periodontal health. A recent study highlighted an increased risk of periodontitis linked to elevated probing pocket depths and clinical attachment loss in patients receiving thyroid hormone therapy [39]. This connection suggests a potential area for further investigation, particularly regarding the role of thyroid hormones in periodontal disease progression.

### Strengths, limitations and way forward

The strengths of this manuscript lie in its comprehensive analysis of drug-associated gingival disorders using a large dataset from the USFDA AERS, which enhances the generalizability of findings across diverse populations. By employing both frequentist and Bayesian disproportionality analysis methods, the study robustly identifies significant associations between various medications and specific gingival disorders, filling a critical gap in the literature. Volcano plots were generated for each gum disorder to identify the drugs with the strongest signals based on the statistical significance and magnitude of association. However, the limitations include the reliance on spontaneous reporting data, which can introduce biases such as underreporting and variability in reporting practices [40]. Additionally, the study does not establish causal relationships, as adverse events are reported based on clinical judgment without controlled studies. Future real-world studies should focus on obtaining more details on the data on time to onset, dechallenge, rechallenge, dosage, and comorbidities for case-by-case analysis and for establishing causal relationships. The OpenVigil platform, despite being one of the two widely used open-access tools for identifying potential safety signals through disproportionality analysis in pharmacovigilance studies, has faced criticism for offering only partial transparency in its data cleaning and pre-processing methods [41]. The deduplication procedure in this study followed USFDA recommendations, aligning with methodologies utilized in several other studies to ensure the inclusion of only unique reports based on the Case IDs and ICSRs which doesn’t ensure a complete deduplication. Emerging deduplication approaches, including the application of large language models, are being explored to further refine the process [42]. Future research could benefit from incorporating such innovative techniques to enhance deduplication accuracy and efficiency. Furthermore, we restricted our data collection to the USFDA database, which resulted in many reports originating from the USA. Consequently, it remains unclear whether geographical variations exist in the occurrence of drug-associated gingival disorders. The findings of this pharmacovigilance study have significant implications for both clinical practice and drug safety monitoring systems. From a clinical perspective, healthcare providers should implement systematic oral health screening and more frequent dental monitoring for patients prescribed high-risk medications, particularly anti-osteoporotic drugs, anti-thrombotics, calcium channel blockers, and immunosuppressants. This necessitates a more integrated approach to patient care, where dental professionals are actively involved in medication management decisions and regular monitoring. Healthcare providers should establish baseline dental documentation before initiating these medications and develop individualized dental care protocols for patients on multiple high-risk drugs. Patient education becomes crucial, focusing on medication-specific risks, early warning signs of gingival complications, and the importance of maintaining optimal oral hygiene. From a pharmacovigilance perspective, the findings call for enhanced adverse event reporting systems that specifically incorporate dental parameters and standardized terminology for gingival adverse effects. This should be supported by strengthened collaboration between dental professionals and pharmacovigilance centers. The development of specific algorithms for detecting gingival adverse effects and risk stratification tools for medication-induced gingival disorders is essential. Regulatory authorities should consider updating product information to reflect newly identified gingival risks and implement targeted risk minimization measures for high-risk medications. Additionally, guidelines for managing drug-induced gingival complications should be developed, and mandatory dental monitoring could be considered for certain drug classes. These implications underscore the need for a more comprehensive approach to medication safety that explicitly considers oral health outcomes in both clinical practice and pharmacovigilance systems. Also, future research should focus on longitudinal studies to explore the causal mechanisms underlying these associations and the impact of drug-induced gingival disorders on patients’ oral health and medication adherence. Moreover, incorporating patient-reported outcomes and conducting interdisciplinary studies with dental professionals could further elucidate the clinical significance of these findings and improve patient care strategies.

## Conclusion

In conclusion, this study underscores the significant associations between various medications and drug-associated gingival disorders, revealing a complex interplay between pharmacological agents and oral health. The findings highlight the need for increased awareness among healthcare providers regarding the potential oral side effects of commonly prescribed drugs, particularly anti-osteoporotic agents, anti-thrombotics, calcium channel blockers, and immunosuppressants. Given the varied presentations of gingival disorders, including atrophy, hypertrophy, discoloration, and bleeding, it is imperative that clinicians remain vigilant in monitoring oral health in patients undergoing treatment with these medications. Furthermore, this study identifies critical gaps in current clinical guidelines concerning dental monitoring for patients on specific drug therapies. Future research should focus on elucidating the underlying mechanisms of drug-induced gingival disorders and developing interdisciplinary strategies that promote proactive dental care and comprehensive management of patients, ultimately enhancing patient outcomes in drug-induced periodontal management. The emergent signals identified, especially concerning finasteride, levothyroxine and COVID-19 vaccines, warrant further investigation in prospective studies to elucidate the underlying mechanisms and clinical implications of these relationships. Interdisciplinary collaboration between medical and dental practitioners will be essential in developing comprehensive management strategies for patients at risk of drug-associated gum disorders.

## Supplementary information

Supplementary Information

## Data Availability

The datasets generated during and/or analyzed during the current study are available from the corresponding author on reasonable request.
