# Supplementary material for: Drug-associated gingival disorders: a retrospective pharmacovigilance assessment using disproportionality analysis
Source: BDJ Open. 2025 Mar 11;11:24. doi: 10.1038/s41405-024-00291-8 (PMC11897221; doi:10.1038/s41405-024-00291-8)
Supplement: Supplementary file 1 — Supplementary Information [file 41405_2024_291_MOESM1_ESM.docx]

**Supplemental Table 1. Signal detection measures for drug-associated gingival bleeding.**

| **ATC** | **Drug** | **PRR** | **χ2** | **RRR** | **ROR** | **Lower limit of 95% CI of ROR** | **Upper limit of 95% CI of ROR** | **IC025** | **EBGM05** | **Number of cases** |
| --- | --- | --- | --- | --- | --- | --- | --- | --- | --- | --- |
| Agents acting on the renin-angiotensin system | Losartan | 2.1 | 50.4 | 2.0 | 2.1 | 1.7 | 2.5 | 0.8 | 1.7 | 97 |
|  | Ramipril | 2.1 | 42.1 | 2.1 | 2.1 | 1.7 | 2.7 | 0.9 | 1.7 | 74 |
|  | Candesartan | 4.5 | 154.6 | 4.5 | 4.5 | 3.5 | 5.9 | 1.7 | 3.5 | 58 |
|  | Enalapril | 2.4 | 27.2 | 2.4 | 2.4 | 1.7 | 3.3 | 0.9 | 1.7 | 36 |
|  | Irbesartan | 2.4 | 26.3 | 2.4 | 2.4 | 1.7 | 3.4 | 0.9 | 1.7 | 34 |
| All other therapeutic products | Leucovorin | 2.5 | 32.3 | 2.5 | 2.5 | 1.8 | 3.4 | 1 | 1.8 | 38 |
| Analgesics | Dextropropoxyphene | 2.7 | 5.0 | 2.7 | 2.7 | 1.2 | 6.1 | 0.7 | 1.2 | 6 |
|  | Dihydrocodeine | 3.1 | 5.0 | 3.1 | 3.1 | 1.3 | 7.4 | 0.7 | 1.3 | 5 |
| Antibacterials for systemic use | Clarithromycin | 2.3 | 19.9 | 2.3 | 2.3 | 1.6 | 3.4 | 0.8 | 1.6 | 28 |
|  | Cephalexin | 2.3 | 12.2 | 2.3 | 2.3 | 1.5 | 3.7 | 0.8 | 1.5 | 18 |
|  | Cefpodoxime | 5.5 | 7.0 | 5.5 | 5.5 | 1.8 | 17.1 | 0.8 | 1.8 | 3 |
| Antidiarrheals, intestinal anti-inflammatory/anti-infective agents | Nystatin | 2.5 | 8.1 | 2.5 | 2.5 | 1.4 | 4.4 | 0.7 | 1.4 | 11 |
|  | Loperamide oxide | 178.5 | 367.0 | 178.5 | 196.3 | 59.9 | 643.4 | 2.3 | 54.4 | 3 |
| Antiemetics and Antinauseants | Dronabinol | 3.3 | 5.8 | 3.3 | 3.3 | 1.4 | 7.9 | 0.7 | 1.4 | 5 |
| Antiepileptics | Topiramate | 2.0 | 27.1 | 2.0 | 2.0 | 1.6 | 2.7 | 0.8 | 1.6 | 53 |
|  | Carbamazepine | 2.4 | 38.9 | 2.4 | 2.4 | 1.8 | 3.2 | 1 | 1.8 | 49 |
|  | Phenytoin | 3.3 | 67.8 | 3.3 | 3.3 | 2.5 | 4.4 | 1.3 | 2.4 | 44 |
|  | Primidone | 5.8 | 47.4 | 5.8 | 5.9 | 3.4 | 10.1 | 1.5 | 3.4 | 13 |
| Antifungals for dermatological use | Amorolfine | 39.8 | 78.0 | 39.8 | 40.6 | 12.9 | 127.4 | 1.7 | 12.7 | 3 |
| Antigout preparations | Allopurinol | 2.8 | 106.9 | 2.8 | 2.9 | 2.3 | 3.5 | 1.2 | 2.3 | 92 |
| Antihemorrhagics | Eltrombopag | 14.1 | 1071.9 | 14.0 | 14.2 | 11.6 | 17.5 | 3.1 | 11.3 | 90 |
|  | Coagulation factor VIII | 20.2 | 1031.3 | 20.0 | 20.4 | 15.7 | 26.5 | 3.3 | 15.5 | 58 |
|  | Emicizumab | 17.9 | 744.2 | 17.8 | 18.1 | 13.6 | 24.0 | 3.1 | 13.4 | 48 |
|  | Romiplostim | 9.9 | 374.8 | 9.9 | 10.0 | 7.5 | 13.3 | 2.5 | 7.4 | 48 |
|  | Coagulation factor IX | 18.8 | 268.0 | 18.7 | 18.9 | 11.7 | 30.6 | 2.6 | 11.5 | 17 |
|  | Tranexamic acid | 7.6 | 67.8 | 7.6 | 7.6 | 4.4 | 13.1 | 1.7 | 4.4 | 13 |
|  | Aminocaproic acid | 44.2 | 463.7 | 44.1 | 45.2 | 25.5 | 80.1 | 3.1 | 24.9 | 12 |
|  | Efmoroctocog alfa | 11.9 | 89.2 | 11.9 | 12.0 | 6.4 | 22.3 | 1.9 | 6.4 | 10 |
|  | Avatrombopag | 14.3 | 98.2 | 14.3 | 14.4 | 7.5 | 27.7 | 2 | 7.4 | 9 |
|  | Von Willebrand factor | 35.5 | 268.2 | 35.5 | 36.2 | 18.7 | 69.9 | 2.7 | 18.3 | 9 |
|  | Fostamatinib | 3.7 | 11.0 | 3.7 | 3.7 | 1.7 | 7.7 | 0.9 | 1.7 | 7 |
|  | Albutrepenonacog alfa | 30.3 | 141.9 | 30.3 | 30.8 | 13.7 | 68.9 | 2.2 | 13.5 | 6 |
|  | Eftrenonacog alfa | 9.9 | 31.7 | 9.9 | 10.0 | 4.1 | 24.0 | 1.4 | 4.1 | 5 |
|  | Turoctocog alfa | 22.6 | 42.2 | 22.6 | 22.8 | 7.3 | 71.3 | 1.4 | 7.2 | 3 |
|  | Beroctocog alfa | 19.4 | 35.5 | 19.4 | 19.6 | 6.3 | 61.0 | 1.4 | 6.2 | 3 |
| Antihistamines for systemic use | Dimenhydrinate | 3.9 | 12.6 | 3.9 | 4.0 | 1.9 | 8.3 | 0.9 | 1.9 | 7 |
|  | Bilastine | 4.7 | 8.2 | 4.7 | 4.7 | 1.8 | 12.5 | 0.8 | 1.8 | 4 |
| Antihypertensives | Doxazosin | 4.6 | 115.7 | 4.6 | 4.7 | 3.4 | 6.3 | 1.6 | 3.4 | 42 |
|  | Riociguat | 2.3 | 15.3 | 2.3 | 2.3 | 1.5 | 3.5 | 0.8 | 1.5 | 22 |
| Antiinflammatory and antirheumatic products | Glucosamine | 4.6 | 27.0 | 4.5 | 4.6 | 2.5 | 8.2 | 1.2 | 2.5 | 11 |
|  | Indomethacin | 2.3 | 5.1 | 2.3 | 2.3 | 1.2 | 4.3 | 0.6 | 1.2 | 9 |
|  | Mefenamic acid | 7.7 | 34.3 | 7.7 | 7.7 | 3.7 | 16.2 | 1.4 | 3.7 | 7 |
|  | Piroxicam | 3.3 | 7.3 | 3.3 | 3.3 | 1.5 | 7.3 | 0.7 | 1.4 | 6 |
| Antimycobacterials | Isoniazid | 3.6 | 37.3 | 3.6 | 3.6 | 2.4 | 5.6 | 1.2 | 2.4 | 21 |
|  | Ethambutol | 3.4 | 23.0 | 3.4 | 3.4 | 2.0 | 5.6 | 1.1 | 2 | 15 |
|  | Dapsone | 3.3 | 10.6 | 3.3 | 3.3 | 1.7 | 6.6 | 0.9 | 1.7 | 8 |
| Antineoplastic agents | Bevacizumab | 3.0 | 148.3 | 3.0 | 3.1 | 2.5 | 3.7 | 1.3 | 2.5 | 111 |
|  | Palbociclib | 2.3 | 56.3 | 2.3 | 2.3 | 1.8 | 2.9 | 1 | 1.8 | 80 |
|  | Cabozantinib | 4.8 | 199.6 | 4.8 | 4.8 | 3.8 | 6.1 | 1.8 | 3.8 | 68 |
|  | Niraparib | 8.2 | 418.0 | 8.1 | 8.2 | 6.5 | 10.4 | 2.4 | 6.4 | 68 |
|  | Ibrutinib | 2.6 | 59.2 | 2.6 | 2.6 | 2.0 | 3.3 | 1.1 | 2 | 64 |
|  | Oxaliplatin | 2.5 | 49.8 | 2.5 | 2.6 | 2.0 | 3.3 | 1 | 1.9 | 55 |
|  | Ruxolitinib | 2.2 | 31.7 | 2.2 | 2.2 | 1.7 | 2.9 | 0.9 | 1.7 | 51 |
|  | Sunitinib | 4.8 | 121.0 | 4.8 | 4.8 | 3.5 | 6.5 | 1.7 | 3.5 | 42 |
|  | Sorafenib | 5.0 | 107.2 | 5.0 | 5.0 | 3.6 | 7.0 | 1.7 | 3.5 | 35 |
|  | Lenvatinib | 2.9 | 35.5 | 2.9 | 2.9 | 2.0 | 4.2 | 1.1 | 2 | 30 |
|  | Irinotecan | 2.2 | 16.3 | 2.2 | 2.2 | 1.5 | 3.3 | 0.8 | 1.5 | 26 |
|  | Ripretinib | 16.9 | 372.8 | 16.9 | 17.1 | 11.6 | 25.1 | 2.8 | 11.5 | 26 |
|  | Azacitidine | 3.1 | 33.8 | 3.1 | 3.1 | 2.1 | 4.6 | 1.1 | 2.1 | 25 |
|  | Ribociclib | 2.4 | 18.9 | 2.4 | 2.4 | 1.6 | 3.6 | 0.8 | 1.6 | 25 |
|  | Nilotinib | 2.0 | 10.2 | 2.0 | 2.0 | 1.3 | 3.1 | 0.7 | 1.3 | 22 |
|  | Temozolomide | 2.2 | 10.1 | 2.2 | 2.2 | 1.4 | 3.4 | 0.7 | 1.4 | 18 |
|  | Trametinib | 2.1 | 9.5 | 2.1 | 2.1 | 1.3 | 3.4 | 0.7 | 1.3 | 18 |
|  | Hydroxyurea | 2.9 | 19.6 | 2.9 | 2.9 | 1.8 | 4.7 | 1 | 1.8 | 17 |
|  | Trastuzumab emtansine | 14.8 | 191.3 | 14.7 | 14.9 | 9.1 | 24.3 | 2.4 | 9 | 16 |
|  | Avapritinib | 3.7 | 23.4 | 3.7 | 3.7 | 2.2 | 6.5 | 1.1 | 2.2 | 13 |
|  | Regorafenib | 3.7 | 23.4 | 3.7 | 3.7 | 2.2 | 6.5 | 1.1 | 2.2 | 13 |
|  | Decitabine | 6.9 | 44.3 | 6.8 | 6.9 | 3.7 | 12.8 | 1.5 | 3.7 | 10 |
|  | Gefitinib | 2.5 | 5.9 | 2.5 | 2.5 | 1.3 | 5.1 | 0.7 | 1.3 | 8 |
|  | Cladribine | 2.5 | 5.1 | 2.5 | 2.5 | 1.2 | 5.3 | 0.6 | 1.2 | 7 |
|  | Anagrelide | 5.5 | 14.1 | 5.5 | 5.5 | 2.3 | 13.2 | 1 | 2.3 | 5 |
|  | Ramucirumab | 3.1 | 5.1 | 3.1 | 3.1 | 1.3 | 7.4 | 0.7 | 1.3 | 5 |
|  | Gemtuzumab ozogamicin | 3.7 | 5.4 | 3.7 | 3.7 | 1.4 | 9.9 | 0.7 | 1.4 | 4 |
|  | Zanubrutinib | 5.0 | 9.2 | 5.0 | 5.0 | 1.9 | 13.4 | 0.9 | 1.9 | 4 |
|  | Duvelisib | 14.6 | 25.6 | 14.6 | 14.7 | 4.7 | 45.7 | 1.2 | 4.7 | 3 |
|  | Fruquintinib | 9.8 | 15.7 | 9.8 | 9.8 | 3.2 | 30.5 | 1.1 | 3.1 | 3 |
|  | Inotuzumab ozogamicin | 4.0 | 4.1 | 4.0 | 4.0 | 1.3 | 12.5 | 0.6 | 1.3 | 3 |
|  | Omacetaxine mepesuccinate | 10.7 | 17.5 | 10.6 | 10.7 | 3.4 | 33.3 | 1.1 | 3.4 | 3 |
| Antiprotozoals | Quinine | 2.9 | 5.9 | 2.9 | 3.0 | 1.3 | 6.6 | 0.7 | 1.3 | 6 |
| Antiseptics and Disinfectants | Triclosan | 21.1 | 95.4 | 21.0 | 21.3 | 9.5 | 47.6 | 2 | 9.4 | 6 |
| Antithrombotic agents | Enoxaparin sodium | 5.1 | 9.3 | 5.1 | 5.1 | 1.9 | 13.5 | 0.9 | 1.9 | 4 |
|  | Rivaroxaban | 9.1 | 3351.7 | 8.4 | 9.1 | 8.3 | 10.0 | 2.8 | 7.7 | 507 |
|  | Apixaban | 3.9 | 651.7 | 3.8 | 3.9 | 3.5 | 4.4 | 1.7 | 3.4 | 315 |
|  | Warfarin | 5.1 | 893.3 | 4.9 | 5.1 | 4.5 | 5.7 | 2 | 4.3 | 288 |
|  | Clopidogrel | 4.8 | 685.5 | 4.7 | 4.8 | 4.2 | 5.5 | 2 | 4.1 | 237 |
|  | Acetylsalicylic acid | 2.5 | 118.7 | 2.4 | 2.5 | 2.1 | 2.9 | 1.1 | 2.1 | 139 |
|  | Dabigatran etexilate | 5.7 | 506.0 | 5.6 | 5.7 | 4.8 | 6.7 | 2.1 | 4.7 | 136 |
|  | Alteplase | 17.8 | 1131.6 | 17.7 | 18.0 | 14.3 | 22.7 | 3.3 | 14 | 73 |
|  | Enoxaparin | 2.7 | 51.5 | 2.7 | 2.7 | 2.0 | 3.5 | 1.1 | 2 | 51 |
|  | Heparin | 3.1 | 67.0 | 3.1 | 3.1 | 2.4 | 4.2 | 1.2 | 2.3 | 48 |
|  | Ticagrelor | 4.5 | 126.0 | 4.5 | 4.5 | 3.4 | 6.0 | 1.6 | 3.4 | 48 |
|  | Acenocoumarol | 15.0 | 414.7 | 14.9 | 15.1 | 10.7 | 21.3 | 2.8 | 10.6 | 33 |
|  | Caplacizumab | 54.2 | 1352.1 | 54.0 | 55.7 | 38.0 | 81.7 | 3.9 | 36.8 | 27 |
|  | Prasugrel | 5.2 | 66.7 | 5.2 | 5.2 | 3.4 | 8.0 | 1.5 | 3.4 | 21 |
|  | Edoxaban tosylate | 4.8 | 38.8 | 4.8 | 4.8 | 2.9 | 8.2 | 1.3 | 2.9 | 14 |
|  | Eptifibatide | 14.8 | 140.6 | 14.8 | 14.9 | 8.4 | 26.3 | 2.2 | 8.4 | 12 |
|  | Fluindione | 9.8 | 86.1 | 9.8 | 9.8 | 5.6 | 17.4 | 1.9 | 5.5 | 12 |
|  | Edoxaban | 8.5 | 65.3 | 8.5 | 8.5 | 4.7 | 15.4 | 1.7 | 4.7 | 11 |
|  | Iloprost | 2.6 | 8.3 | 2.6 | 2.6 | 1.4 | 4.8 | 0.7 | 1.4 | 10 |
|  | Fondaparinux sodium | 4.3 | 19.9 | 4.3 | 4.3 | 2.3 | 8.4 | 1.1 | 2.3 | 9 |
|  | Dipyridamole | 3.0 | 8.5 | 3.0 | 3.0 | 1.5 | 5.9 | 0.8 | 1.5 | 8 |
|  | Cilostazol | 3.2 | 8.7 | 3.2 | 3.2 | 1.5 | 6.8 | 0.8 | 1.5 | 7 |
|  | Tirofiban | 8.1 | 36.8 | 8.1 | 8.1 | 3.9 | 17.1 | 1.4 | 3.9 | 7 |
|  | Phenprocoumon | 3.0 | 6.3 | 3.0 | 3.0 | 1.4 | 6.8 | 0.7 | 1.4 | 6 |
|  | Abciximab | 5.6 | 14.6 | 5.6 | 5.6 | 2.3 | 13.5 | 1 | 2.3 | 5 |
|  | Bivalirudin | 5.7 | 15.1 | 5.7 | 5.8 | 2.4 | 13.8 | 1 | 2.4 | 5 |
|  | Certoparin | 30.2 | 113.5 | 30.2 | 30.7 | 12.7 | 74.2 | 2 | 12.4 | 5 |
|  | Tenecteplase | 6.8 | 14.5 | 6.8 | 6.9 | 2.6 | 18.3 | 1 | 2.6 | 4 |
|  | Streptokinase | 57.8 | 115.4 | 57.7 | 59.5 | 18.9 | 187.7 | 1.9 | 18.3 | 3 |
| Antivirals for systemic use | Ribavirin | 4.4 | 315.4 | 4.3 | 4.4 | 3.7 | 5.2 | 1.7 | 3.6 | 125 |
|  | Telaprevir | 3.6 | 59.9 | 3.6 | 3.6 | 2.5 | 5.0 | 1.3 | 2.5 | 34 |
| Beta-blocking agents | Metoprolol | 2.0 | 84.6 | 2.0 | 2.0 | 1.7 | 2.4 | 0.9 | 1.7 | 169 |
|  | Bisoprolol | 2.4 | 67.6 | 2.4 | 2.4 | 1.9 | 3.0 | 1 | 1.9 | 86 |
|  | Atenolol | 2.3 | 49.0 | 2.3 | 2.3 | 1.8 | 2.9 | 0.9 | 1.8 | 71 |
|  | Carvedilol | 2.3 | 50.0 | 2.3 | 2.3 | 1.8 | 2.9 | 0.9 | 1.8 | 70 |
|  | Propranolol | 2.6 | 44.9 | 2.6 | 2.6 | 1.9 | 3.4 | 1 | 1.9 | 48 |
|  | Sotalol | 5.3 | 78.5 | 5.3 | 5.3 | 3.5 | 7.9 | 1.6 | 3.5 | 24 |
|  | Nebivolol | 2.4 | 14.6 | 2.4 | 2.4 | 1.5 | 3.7 | 0.8 | 1.5 | 20 |
| Calcium channel blockers | Amlodipine | 3.7 | 685.9 | 3.5 | 3.7 | 3.3 | 4.1 | 1.6 | 3.2 | 379 |
|  | Nifedipine | 2.6 | 33.8 | 2.6 | 2.6 | 1.9 | 3.7 | 1 | 1.9 | 35 |
|  | Verapamil | 2.0 | 10.6 | 2.0 | 2.0 | 1.3 | 3.0 | 0.7 | 1.3 | 23 |
|  | Lercanidipine | 2.4 | 6.5 | 2.4 | 2.4 | 1.3 | 4.4 | 0.7 | 1.3 | 10 |
| Cardiac therapy | Amiodarone | 2.2 | 23.9 | 2.2 | 2.2 | 1.6 | 3.0 | 0.8 | 1.6 | 40 |
|  | Digoxin | 2.1 | 18.7 | 2.1 | 2.1 | 1.5 | 2.9 | 0.8 | 1.5 | 36 |
|  | Nitroglycerin | 2.1 | 14.5 | 2.1 | 2.1 | 1.5 | 3.1 | 0.7 | 1.4 | 26 |
|  | Dronedarone | 4.8 | 41.0 | 4.8 | 4.8 | 2.9 | 7.9 | 1.4 | 2.9 | 15 |
|  | Flecainide | 3.2 | 19.4 | 3.2 | 3.2 | 1.9 | 5.5 | 1 | 1.9 | 14 |
|  | Isosorbide dinitrate | 4.1 | 18.3 | 4.1 | 4.1 | 2.2 | 8.0 | 1.1 | 2.1 | 9 |
| Corticosteroids for systemic use | Methylprednisolone | 2.1 | 9.1 | 2.1 | 2.1 | 1.3 | 3.4 | 0.7 | 1.3 | 17 |
|  | Cortisone | 2.4 | 5.3 | 2.4 | 2.4 | 1.2 | 4.8 | 0.6 | 1.2 | 8 |
| Diagnostic radiopharmaceuticals | Sodium iodide I^131^ | 13.2 | 33.8 | 13.2 | 13.3 | 5.0 | 35.6 | 1.4 | 4.9 | 4 |
| Diuretics | Indapamide | 2.8 | 16.5 | 2.8 | 2.8 | 1.7 | 4.5 | 0.9 | 1.7 | 16 |
| Drugs for acid related disorders | Magnesium oxide | 2.4 | 13.0 | 2.4 | 2.4 | 1.5 | 3.9 | 0.8 | 1.5 | 17 |
|  | Cimetidine | 2.8 | 5.4 | 2.8 | 2.8 | 1.3 | 6.3 | 0.7 | 1.3 | 6 |
| Drugs for constipation | Lactulose | 3.2 | 28.4 | 3.2 | 3.2 | 2.1 | 5.0 | 1.1 | 2.1 | 20 |
| Drugs for obstructive airway diseases | Beclometasone dipropionate | 4.2 | 24.0 | 4.2 | 4.2 | 2.3 | 7.7 | 1.2 | 2.3 | 11 |
| Drugs used for treatment of bone diseases | Alendronic acid | 14.5 | 2756.6 | 14.0 | 14.6 | 12.8 | 16.6 | 3.3 | 12.3 | 229 |
|  | Zoledronic acid | 4.0 | 217.9 | 3.9 | 4.0 | 3.3 | 4.9 | 1.6 | 3.2 | 100 |
|  | Ibandronate | 3.5 | 59.1 | 3.5 | 3.5 | 2.5 | 4.9 | 1.3 | 2.5 | 35 |
|  | Pamidronic acid | 18.9 | 454.5 | 18.8 | 19.0 | 13.1 | 27.7 | 2.9 | 12.9 | 28 |
|  | Risedronic acid | 5.4 | 71.0 | 5.4 | 5.4 | 3.5 | 8.3 | 1.5 | 3.5 | 21 |
| Endocrine therapy | Letrozole | 2.7 | 51.6 | 2.7 | 2.7 | 2.0 | 3.6 | 1.1 | 2 | 50 |
|  | Fulvestrant | 2.7 | 25.4 | 2.7 | 2.7 | 1.8 | 3.9 | 1 | 1.8 | 26 |
|  | Tamoxifen | 3.8 | 45.7 | 3.7 | 3.8 | 2.5 | 5.6 | 1.3 | 2.5 | 24 |
|  | Anastrozole | 2.1 | 11.5 | 2.1 | 2.1 | 1.4 | 3.3 | 0.7 | 1.4 | 21 |
|  | Exemestane | 2.1 | 6.4 | 2.1 | 2.1 | 1.2 | 3.6 | 0.6 | 1.2 | 13 |
|  | Flutamide | 12.7 | 32.2 | 12.7 | 12.8 | 4.8 | 34.1 | 1.4 | 4.7 | 4 |
| Immunostimulants | Peginterferon alfa-2a | 5.4 | 341.5 | 5.3 | 5.4 | 4.4 | 6.6 | 2 | 4.4 | 98 |
|  | Peginterferon alfa-2b | 5.2 | 105.4 | 5.2 | 5.3 | 3.7 | 7.4 | 1.7 | 3.7 | 32 |
| Immunosuppressants | Cyclosporine | 2.5 | 73.4 | 2.5 | 2.5 | 2.0 | 3.2 | 1.1 | 2.1 | 81 |
|  | Alemtuzumab | 7.3 | 188.7 | 7.3 | 7.3 | 5.3 | 10.2 | 2.1 | 5.2 | 36 |
|  | Voclosporin | 3.7 | 11.2 | 3.7 | 3.7 | 1.8 | 7.8 | 0.8 | 1.8 | 7 |
|  | Teprotumumab | 3.9 | 10.1 | 3.9 | 3.9 | 1.7 | 8.7 | 0.9 | 1.7 | 6 |
| Lipid modifying agents | Rosuvastatin calcium | 2.1 | 12.0 | 2.1 | 2.1 | 1.4 | 3.2 | 0.7 | 1.4 | 22 |
|  | Gemfibrozil | 3.9 | 27.7 | 3.9 | 3.9 | 2.3 | 6.7 | 1.2 | 2.3 | 14 |
|  | Omega-3 fatty acids | 3.3 | 7.7 | 3.3 | 3.4 | 1.5 | 7.5 | 0.8 | 1.5 | 6 |
| Mineral supplements | Zinc | 2.2 | 4.0 | 2.2 | 2.2 | 1.1 | 4.4 | 0.6 | 1.1 | 8 |
|  | Potassium nitrate | 34.8 | 197.3 | 34.8 | 35.4 | 16.8 | 74.9 | 2.4 | 16.5 | 7 |
|  | Calcium ascorbate | 47.5 | 228.7 | 47.5 | 48.7 | 21.7 | 109.5 | 2.5 | 21.1 | 6 |
| Not classified | Iguratimod | 9.9 | 16.0 | 9.9 | 10.0 | 3.2 | 31.0 | 1.1 | 3.2 | 3 |
| Ophthalmologicals | Acetazolamide | 3.6 | 10.8 | 3.6 | 3.6 | 1.7 | 7.6 | 0.9 | 1.7 | 7 |
| Other hematological agents | Lanadelumab | 4.2 | 11.6 | 4.2 | 4.2 | 1.9 | 9.4 | 0.9 | 1.9 | 6 |
| Other nervous system drugs | Varenicline | 3.5 | 217.1 | 3.5 | 3.5 | 2.9 | 4.2 | 1.5 | 2.9 | 125 |
|  | Nicotine | 2.8 | 77.8 | 2.8 | 2.8 | 2.2 | 3.5 | 1.2 | 2.2 | 70 |
|  | Betahistine | 6.5 | 32.2 | 6.5 | 6.6 | 3.3 | 13.1 | 1.4 | 3.3 | 8 |
| Pituitary and hypothalamic hormones and analogues | Desmopressin | 2.8 | 6.1 | 2.7 | 2.8 | 1.3 | 5.8 | 0.7 | 1.3 | 7 |
| Psychoanaleptics | Milnacipran | 3.8 | 11.9 | 3.8 | 3.8 | 1.8 | 8.0 | 0.9 | 1.8 | 7 |
|  | Agomelatine | 10.1 | 32.2 | 10.1 | 10.1 | 4.2 | 24.4 | 1.4 | 4.2 | 5 |
| Psycholeptics | Lormetazepam | 3.2 | 5.5 | 3.2 | 3.2 | 1.3 | 7.7 | 0.7 | 1.3 | 5 |
|  | Clorazepic acid | 5.1 | 9.3 | 5.1 | 5.1 | 1.9 | 13.5 | 0.9 | 1.9 | 4 |
| Sex hormones and modulators of the genital system | Danazol | 14.3 | 86.3 | 14.3 | 14.4 | 7.2 | 28.9 | 1.9 | 7.1 | 8 |
|  | Estradiol valerate | 17.5 | 77.4 | 17.4 | 17.6 | 7.9 | 39.4 | 1.8 | 7.8 | 6 |
|  | Megestrol | 2.8 | 4.1 | 2.8 | 2.8 | 1.2 | 6.7 | 0.6 | 1.2 | 5 |
| Stomatological preparations | Sodium fluoride | 20.2 | 362.7 | 20.1 | 20.4 | 13.2 | 31.3 | 2.8 | 13.1 | 21 |
|  | Chlorhexidine | 4.9 | 27.3 | 4.9 | 4.9 | 2.6 | 9.2 | 1.2 | 2.6 | 10 |
|  | Stannous fluoride | 71.8 | 628.1 | 71.6 | 74.4 | 39.6 | 140.0 | 3.3 | 38.1 | 10 |
|  | Clotrimazole | 3.2 | 11.3 | 3.2 | 3.2 | 1.7 | 6.1 | 0.9 | 1.7 | 9 |
| Topical products for joint and muscular pain | Loxoprofen | 2.8 | 6.4 | 2.8 | 2.8 | 1.3 | 5.9 | 0.7 | 1.3 | 7 |
| Urologicals | Finasteride | 2.1 | 15.1 | 2.1 | 2.1 | 1.5 | 3.1 | 0.7 | 1.5 | 27 |
|  | Fesoterodine | 2.6 | 7.1 | 2.6 | 2.6 | 1.3 | 4.9 | 0.7 | 1.3 | 9 |
| Vaccines | Moderna covid-19 vaccine | 6.8 | 9.6 | 6.8 | 6.8 | 2.2 | 21.2 | 0.9 | 2.2 | 3 |
| Vitamins | Ascorbic acid | 3.3 | 4.2 | 3.3 | 3.3 | 1.2 | 8.7 | 0.6 | 1.2 | 4 |
|  | Cholecalciferol | 2.2 | 90.9 | 2.2 | 2.2 | 1.9 | 2.6 | 9.8 | 1.8 | 146 |
|  | Folic acid | 2.2 | 76.7 | 2.2 | 2.2 | 1.8 | 2.7 | 0.9 | 1.8 | 119 |
|  | Vitamin c | 2.5 | 51.3 | 2.5 | 2.5 | 1.9 | 3.3 | 1 | 1.9 | 58 |
|  | Cyanocobalamin | 2.4 | 38.6 | 2.4 | 2.4 | 1.8 | 3.1 | 0.9 | 1.8 | 51 |
|  | Pyridoxine | 2.3 | 32.6 | 2.3 | 2.3 | 1.7 | 3.1 | 0.9 | 1.7 | 47 |
|  | Vitamin B12 | 2.1 | 16.2 | 2.1 | 2.1 | 1.5 | 3.1 | 0.8 | 1.5 | 29 |
|  | Vitamin E | 2.7 | 13.7 | 2.7 | 2.7 | 1.6 | 4.6 | 0.9 | 1.6 | 14 |
|  | Calcitriol | 2.4 | 6.6 | 2.4 | 2.4 | 1.3 | 4.4 | 0.7 | 1.3 | 10 |
|  | Tocopherol | 12.6 | 53.1 | 12.6 | 12.7 | 5.7 | 28.3 | 1.6 | 5.6 | 6 |

ATC: Anatomical Therapeutic Chemical; PRR: Proportional reporting ratio; χ2: Chi square; RRR: Relative reporting ratio; ROR: Reporting odds ratio; IC: Information component; and EBGM: Empirical Bayes geometric mean.

**Supplemental Table 2. Signal detection measures for drug-associated gingival hypertrophy.**

| **ATC** | **Drug** | **PRR** | **χ2** | **RRR** | **ROR** | **Lower limit of 95% CI of ROR** | **Upper limit of 95% CI of ROR** | **IC025** | **EBGM05** | **Number of reports** |
| --- | --- | --- | --- | --- | --- | --- | --- | --- | --- | --- |
| Agents acting on the renin-angiotensin system | Ramipril | 9.9 | 482.6 | 9.4 | 9.9 | 7.7 | 12.7 | 2.5 | 7.3 | 65 |
|  | Candesartan | 4.0 | 19.2 | 3.9 | 4.0 | 2.1 | 7.4 | 1.1 | 2.1 | 10 |
|  | Benazepril | 4.7 | 16.8 | 4.7 | 4.7 | 2.2 | 9.9 | 1.1 | 2.2 | 7 |
|  | Telmisartan | 3.8 | 11.7 | 3.8 | 3.8 | 1.8 | 8.0 | 0.9 | 1.8 | 7 |
|  | Captopril | 12.8 | 53.6 | 12.7 | 12.8 | 5.7 | 28.5 | 1.6 | 5.7 | 6 |
|  | Enalapril | 7.1 | 103.0 | 7.0 | 7.1 | 4.6 | 11.0 | 1.8 | 4.6 | 21 |
|  | Losartan | 2.2 | 11.0 | 2.1 | 2.2 | 1.4 | 3.4 | 0.7 | 1.4 | 20 |
|  | Irbesartan | 5.1 | 41.2 | 5.0 | 5.1 | 3.0 | 8.6 | 1.4 | 3.0 | 14 |
| Antianemic preparations | Epoetin alfa | 4.7 | 11.2 | 4.7 | 4.7 | 2.0 | 11.4 | 0.9 | 2.0 | 5 |
|  | Erythropoietin | 2.9 | 4.5 | 2.9 | 2.9 | 1.2 | 7.0 | 0.6 | 1.2 | 5 |
| Antibacterials for systemic use | Sulfamethoxazole | 2.5 | 11.6 | 2.5 | 2.5 | 1.5 | 4.1 | 0.8 | 1.5 | 15 |
|  | Trimethoprim | 2.1 | 7.2 | 2.1 | 2.1 | 1.3 | 3.6 | 0.6 | 1.2 | 14 |
|  | Cephalexin | 3.9 | 10.3 | 3.9 | 3.9 | 1.8 | 8.8 | 0.9 | 1.8 | 6 |
|  | Erythromycin | 3.8 | 5.8 | 3.8 | 3.8 | 1.4 | 10.3 | 0.7 | 1.4 | 4 |
| Antiepileptics | Phenytoin | 65.5 | 8436.9 | 57.7 | 65.9 | 55.6 | 78.1 | 4.9 | 48.7 | 152 |
|  | Valproic acid | 9.9 | 352.1 | 9.5 | 9.9 | 7.4 | 13.2 | 2.4 | 7.1 | 47 |
|  | Levetiracetam | 5.7 | 149.2 | 5.5 | 5.7 | 4.2 | 7.8 | 1.8 | 4.1 | 41 |
|  | Topiramate | 6.8 | 158.2 | 6.6 | 6.8 | 4.8 | 9.6 | 1.9 | 4.7 | 34 |
|  | Phenobarbital | 26.6 | 603.3 | 26.1 | 26.7 | 18.1 | 39.4 | 3.2 | 17.7 | 26 |
|  | Carbamazepine | 6.3 | 105.2 | 6.2 | 6.4 | 4.3 | 9.4 | 1.8 | 4.2 | 25 |
|  | Lamotrigine | 2.7 | 20.0 | 2.6 | 2.7 | 1.7 | 4.1 | 0.9 | 1.7 | 21 |
|  | Oxcarbazepine | 3.7 | 10.9 | 3.6 | 3.7 | 1.7 | 7.7 | 0.9 | 1.7 | 7 |
|  | Primidone | 16.1 | 83.9 | 16.0 | 16.1 | 7.7 | 33.8 | 1.9 | 7.6 | 7 |
|  | Divalproex | 4.3 | 12.1 | 4.3 | 4.3 | 1.9 | 9.6 | 0.9 | 1.9 | 6 |
|  | Vigabatrin | 5.5 | 14.1 | 5.5 | 5.5 | 2.3 | 13.2 | 1.0 | 2.3 | 5 |
|  | Zonisamide | 4.7 | 8.3 | 4.7 | 4.7 | 1.8 | 12.6 | 0.8 | 1.8 | 4 |
| Antigout preparations | Colchicine | 4.4 | 12.6 | 4.4 | 4.4 | 2.0 | 9.9 | 1.0 | 2.0 | 6 |
|  | Benzbromarone | 183.0 | 729.4 | 182.3 | 186.4 | 76.8 | 452.2 | 3.1 | 75.1 | 5 |
| Antihypertensives | Doxazosin | 10.2 | 138.3 | 10.1 | 10.2 | 6.4 | 16.3 | 2.1 | 6.3 | 18 |
| Anti-inflammatory and anti-rheumatic products | Indomethacin | 9.0 | 41.6 | 8.9 | 9.0 | 4.3 | 18.8 | 1.5 | 4.2 | 7 |
| Antineoplastic agents | Decitabine | 10.5 | 17.1 | 10.4 | 10.5 | 3.4 | 32.6 | 1.1 | 3.4 | 3 |
| Antipsoriatic drugs | Calcipotriol | 15.2 | 65.5 | 15.1 | 15.2 | 6.8 | 33.9 | 1.8 | 6.8 | 6 |
| Antithrombotic agents | Acenocoumarol | 18.5 | 114.8 | 18.4 | 18.5 | 9.2 | 37.2 | 2.1 | 9.2 | 8 |
| Antivirals for systemic use | Acyclovir | 3.5 | 26.8 | 3.4 | 3.5 | 2.1 | 5.6 | 1.1 | 2.1 | 17 |
| Beta-blocking agents | Atenolol | 5.8 | 130.7 | 5.7 | 5.8 | 4.2 | 8.1 | 1.8 | 4.1 | 35 |
|  | Carvedilol | 2.7 | 15.1 | 2.7 | 2.7 | 1.6 | 4.4 | 0.9 | 1.6 | 16 |
|  | Propranolol | 3.6 | 21.4 | 3.6 | 3.6 | 2.1 | 6.2 | 1.1 | 2.1 | 13 |
|  | Labetalol | 9.3 | 43.7 | 9.3 | 9.3 | 4.4 | 19.6 | 1.5 | 4.4 | 7 |
|  | Sotalol | 5.6 | 14.5 | 5.6 | 5.6 | 2.3 | 13.5 | 1.0 | 2.3 | 5 |
| Bile and liver therapy | Ursodeoxycholic acid | 3.5 | 4.9 | 3.5 | 3.5 | 1.3 | 9.4 | 0.7 | 1.3 | 4 |
| Calcium channel blockers | Amlodipine | 45.1 | 13384.1 | 25.9 | 45.2 | 40.4 | 50.5 | 4.2 | 23.2 | 551 |
|  | Nifedipine | 24.5 | 1283.8 | 23.3 | 24.5 | 18.9 | 31.7 | 3.5 | 18.0 | 61 |
|  | Diltiazem | 7.8 | 178.7 | 7.6 | 7.8 | 5.5 | 11.1 | 2.1 | 5.4 | 32 |
|  | Felodipine | 37.9 | 775.1 | 37.2 | 38.0 | 25.2 | 57.5 | 3.5 | 24.7 | 23 |
|  | Verapamil | 8.1 | 103.2 | 8.0 | 8.1 | 5.1 | 12.9 | 1.9 | 5.0 | 18 |
| Cardiac therapy | Digoxin | 2.6 | 7.5 | 2.6 | 2.6 | 1.4 | 5.1 | 0.7 | 1.4 | 9 |
|  | Mexiletine | 28.4 | 54.2 | 28.4 | 28.5 | 9.2 | 88.6 | 1.6 | 9.1 | 3 |
| Corticosteroids for systemic use | Prednisolone | 5.9 | 219.3 | 5.7 | 5.9 | 4.5 | 7.7 | 1.9 | 4.3 | 58 |
|  | Methylprednisolone | 2.4 | 10.5 | 2.4 | 2.4 | 1.4 | 4.0 | 0.7 | 1.4 | 15 |
| Diuretics | Hydrochlorothiazide | 2.6 | 34.9 | 2.6 | 2.6 | 1.9 | 3.7 | 1.0 | 1.9 | 37 |
|  | Indapamide | 20.7 | 403.6 | 20.3 | 20.7 | 13.7 | 31.3 | 2.9 | 13.4 | 23 |
|  | Bendroflumethiazide | 9.6 | 45.3 | 9.5 | 9.6 | 4.6 | 20.2 | 1.6 | 4.5 | 7 |
| Drugs for acid related disorders | Lansoprazole | 2.4 | 15.5 | 2.3 | 2.4 | 1.5 | 3.6 | 0.8 | 1.5 | 22 |
|  | Rabeprazole | 2.8 | 5.2 | 2.8 | 2.8 | 1.3 | 6.2 | 0.7 | 1.2 | 6 |
| Drugs used for treating bone diseases | Zoledronic acid | 4.1 | 42.5 | 4.0 | 4.1 | 2.6 | 6.3 | 1.3 | 2.6 | 20 |
|  | Risedronic acid | 5.2 | 9.8 | 5.2 | 5.3 | 2.0 | 14.0 | 0.9 | 2.0 | 4 |
| Drugs used in diabetes | Metformin | 2.2 | 35.8 | 2.2 | 2.2 | 1.7 | 2.9 | 0.9 | 1.7 | 57 |
|  | Gliclazide | 11.0 | 134.2 | 10.9 | 11.0 | 6.7 | 18.1 | 2.1 | 6.6 | 16 |
|  | Sitagliptin | 3.1 | 20.2 | 3.1 | 3.1 | 1.9 | 5.1 | 1.0 | 1.9 | 16 |
|  | Glimepiride | 4.7 | 36.8 | 4.7 | 4.7 | 2.8 | 8.0 | 1.3 | 2.8 | 14 |
|  | Glyburide | 4.7 | 22.7 | 4.7 | 4.7 | 2.5 | 9.1 | 1.2 | 2.4 | 9 |
|  | Pioglitazone | 2.5 | 5.0 | 2.5 | 2.5 | 1.2 | 5.3 | 0.6 | 1.2 | 7 |
|  | Vildagliptin | 11.1 | 45.4 | 11.1 | 11.1 | 5.0 | 24.8 | 1.6 | 5.0 | 6 |
| Immunosuppressants | Cyclosporine | 36.4 | 5642.2 | 30.9 | 36.5 | 31.3 | 42.5 | 4.2 | 26.5 | 195 |
|  | Mycophenolate mofetil | 13.0 | 914.3 | 12.1 | 13.0 | 10.5 | 16.1 | 2.9 | 9.8 | 90 |
|  | Tacrolimus | 10.1 | 616.6 | 9.6 | 10.2 | 8.1 | 12.7 | 2.6 | 7.6 | 81 |
|  | Cyclophosphamide | 2.6 | 25.3 | 2.6 | 2.6 | 1.8 | 3.9 | 0.9 | 1.8 | 27 |
|  | Azathioprine | 5.5 | 75.7 | 5.4 | 5.5 | 3.6 | 8.4 | 1.6 | 3.6 | 22 |
|  | Mycophenolic acid | 8.4 | 101.6 | 8.3 | 8.4 | 5.2 | 13.5 | 1.9 | 5.1 | 17 |
|  | Anti-thymocyte immunoglobulin | 41.9 | 435.0 | 41.5 | 42.1 | 23.8 | 74.4 | 3.0 | 23.5 | 12 |
|  | Everolimus | 2.6 | 7.0 | 2.5 | 2.6 | 1.3 | 4.9 | 0.7 | 1.3 | 9 |
|  | Rilonacept | 123.8 | 490.6 | 123.3 | 125.3 | 51.8 | 303.3 | 2.9 | 51.0 | 5 |
|  | Sirolimus | 4.3 | 9.4 | 4.2 | 4.3 | 1.8 | 10.2 | 0.9 | 1.8 | 5 |
| Lipid modifying agents | Atorvastatin | 2.7 | 52.8 | 2.7 | 2.7 | 2.1 | 3.6 | 1.1 | 2.0 | 52 |
|  | Simvastatin | 2.1 | 15.6 | 2.1 | 2.1 | 1.5 | 3.1 | 0.7 | 1.5 | 28 |
| Mineral supplements | Ferrous | 3.1 | 7.9 | 3.1 | 3.1 | 1.5 | 6.5 | 0.8 | 1.5 | 7 |
| Psycholeptics | Clobazam | 15.2 | 285.6 | 14.9 | 15.2 | 10.1 | 23.0 | 2.6 | 9.9 | 23 |
|  | Lithium | 3.7 | 14.9 | 3.7 | 3.7 | 1.9 | 7.1 | 1.0 | 1.9 | 9 |
| Sex hormones and modulators of the genital system | Estradiol valerate | 74.3 | 290.8 | 74.0 | 74.8 | 31.0 | 180.7 | 2.6 | 30.6 | 5 |
| Urologicals | Sildenafil | 2.0 | 7.1 | 2.0 | 2.0 | 1.2 | 3.3 | 0.6 | 1.2 | 16 |
| Vitamins | Calcitriol | 6.0 | 16.2 | 6.0 | 6.0 | 2.5 | 14.5 | 1.1 | 2.5 | 5 |
|  | Alfacalcidol | 7.9 | 11.8 | 7.9 | 7.9 | 2.5 | 24.6 | 1.0 | 2.5 | 3 |
| Not classified | Arotinolol | 464.3 | 1864.2 | 462.5 | 486.8 | 198.1 | 1196.4 | 3.6 | 188.2 | 5 |
|  | Lanolin | 205.6 | 620.6 | 204.9 | 209.8 | 77.8 | 565.7 | 2.8 | 76.0 | 4 |
| Drugs for obstructive airway diseases | Isoproterenol | 93.6 | 189.5 | 93.4 | 94.4 | 30.3 | 294.8 | 2.1 | 29.9 | 3 |

ATC: Anatomical Therapeutic Chemical; PRR: Proportional reporting ratio; χ2: Chi square; RRR: Relative reporting ratio; ROR: Reporting odds ratio; IC: Information component; and EBGM: Empirical Bayes geometric mean.

**Supplemental Table 3. Signal detection measures for drug-associated gingivitis.**

| **ATC** | **Drug** | **PRR** | **χ2** | **RRR** | **ROR** | **Lower limit of 95% CI of ROR** | **Upper limit of 95% CI of ROR** | **IC025** | **EBGM05** | **Number of reports** |
| --- | --- | --- | --- | --- | --- | --- | --- | --- | --- | --- |
| Agents acting on the renin-angiotensin system | Ramipril | 2.4 | 30.7 | 2.4 | 2.4 | 1.7 | 3.2 | 0.9 | 1.7 | 41 |
|  | Candesartan | 4.6 | 76.6 | 4.5 | 4.6 | 3.2 | 6.6 | 1.5 | 3.1 | 29 |
|  | Enalapril | 2.7 | 19.1 | 2.7 | 2.7 | 1.7 | 4.1 | 0.9 | 1.7 | 20 |
| All other therapeutic products | Oxygen | 2.6 | 8.0 | 2.6 | 2.6 | 1.4 | 4.8 | 0.7 | 1.4 | 10 |
|  | Levoleucovorin | 5.3 | 10.0 | 5.3 | 5.3 | 2.0 | 14.1 | 0.9 | 1.9 | 4 |
| Analgesics | Naratriptan | 15.5 | 53.9 | 15.4 | 15.5 | 6.4 | 37.4 | 1.6 | 6.4 | 5 |
| Anesthetics | Lidocaine | 2.9 | 29.4 | 2.8 | 2.9 | 1.9 | 4.2 | 1.0 | 1.9 | 26 |
|  | Prilocaine | 5.8 | 15.5 | 5.8 | 5.8 | 2.4 | 14.1 | 1.1 | 2.4 | 5 |
|  | Articaine | 8.8 | 13.7 | 8.8 | 8.8 | 2.8 | 27.4 | 1.0 | 2.8 | 3 |
| Antianemic preparations | Epoetin alfa | 6.0 | 61.7 | 6.0 | 6.0 | 3.7 | 9.9 | 1.6 | 3.7 | 16 |
|  | Erythropoietin | 3.7 | 28.9 | 3.7 | 3.7 | 2.3 | 6.1 | 1.2 | 2.2 | 16 |
| Antibacterials for systemic use | Amoxicillin | 3.7 | 115.8 | 3.7 | 3.7 | 2.9 | 4.8 | 1.5 | 2.8 | 61 |
|  | Ciprofloxacin | 2.8 | 40.3 | 2.8 | 2.8 | 2.0 | 3.9 | 1.1 | 2.0 | 37 |
|  | Levofloxacin | 2.0 | 12.4 | 2.0 | 2.0 | 1.4 | 3.0 | 0.7 | 1.4 | 26 |
|  | Clavulanic acid | 4.3 | 44.9 | 4.3 | 4.3 | 2.8 | 6.8 | 1.3 | 2.7 | 19 |
|  | Azithromycin | 2.1 | 9.0 | 2.1 | 2.1 | 1.3 | 3.4 | 0.7 | 1.3 | 17 |
|  | Cephalexin | 3.1 | 15.2 | 3.1 | 3.1 | 1.8 | 5.5 | 0.9 | 1.8 | 12 |
|  | Penicillin V | 3.5 | 4.9 | 3.5 | 3.5 | 1.3 | 9.4 | 0.7 | 1.3 | 4 |
| Antiemetics and antinauseants | Granisetron | 6.5 | 36.7 | 6.5 | 6.5 | 3.4 | 12.6 | 1.4 | 3.4 | 9 |
|  | Palonosetron | 6.3 | 21.7 | 6.3 | 6.3 | 2.8 | 14.1 | 1.2 | 2.8 | 6 |
|  | Dolasetron | 20.9 | 38.7 | 20.9 | 21.0 | 6.8 | 65.4 | 1.4 | 6.7 | 3 |
| Antiepileptics | Valproic acid | 2.1 | 13.8 | 2.1 | 2.1 | 1.4 | 3.1 | 0.7 | 1.4 | 26 |
|  | Phenytoin | 3.2 | 29.0 | 3.2 | 3.2 | 2.1 | 4.9 | 1.1 | 2.1 | 21 |
|  | Cannabidiol | 3.8 | 22.2 | 3.8 | 3.8 | 2.2 | 6.7 | 1.1 | 2.2 | 12 |
|  | Rufinamide | 8.4 | 12.8 | 8.3 | 8.4 | 2.7 | 26.0 | 1.0 | 2.7 | 3 |
| Antifungals for dermatological use | Terbinafine | 3.8 | 15.7 | 3.8 | 3.8 | 2.0 | 7.3 | 1.0 | 2.0 | 9 |
|  | Ketoconazole | 3.5 | 9.9 | 3.4 | 3.5 | 1.6 | 7.3 | 0.9 | 1.6 | 7 |
|  | Nystatin | 2.7 | 4.8 | 2.7 | 2.7 | 1.2 | 6.0 | 0.6 | 1.2 | 6 |
| Antihemorrhagics | Eltrombopag olamine | 3.8 | 12.0 | 3.8 | 3.8 | 1.8 | 8.1 | 0.9 | 1.8 | 7 |
| Antihistamines for systemic use | Diphenhydramine | 2.8 | 54.5 | 2.8 | 2.8 | 2.1 | 3.7 | 1.1 | 2.1 | 49 |
| Antihypertensives | Doxazosin | 4.7 | 56.8 | 4.7 | 4.7 | 3.1 | 7.2 | 1.4 | 3.0 | 21 |
|  | Urapidil | 14.8 | 76.4 | 14.7 | 14.8 | 7.0 | 31.1 | 1.8 | 7.0 | 7 |
| Antimycotics for systemic use | Fluconazole | 4.2 | 64.0 | 4.2 | 4.2 | 2.9 | 6.1 | 1.4 | 2.9 | 28 |
|  | Itraconazole | 4.3 | 14.6 | 4.3 | 4.3 | 2.1 | 9.0 | 1.0 | 2.0 | 7 |
| Antineoplastic agents | Bevacizumab | 2.3 | 29.8 | 2.3 | 2.3 | 1.7 | 3.1 | 0.9 | 1.7 | 42 |
|  | Capecitabine | 2.6 | 38.3 | 2.6 | 2.6 | 1.9 | 3.5 | 1.0 | 1.9 | 41 |
|  | Docetaxel | 3.2 | 57.9 | 3.2 | 3.2 | 2.3 | 4.4 | 1.2 | 2.3 | 40 |
|  | Paclitaxel | 2.5 | 32.5 | 2.5 | 2.5 | 1.8 | 3.5 | 1.0 | 1.8 | 38 |
|  | Bortezomib | 2.7 | 34.4 | 2.7 | 2.7 | 1.9 | 3.9 | 1.0 | 1.9 | 33 |
|  | Lenvatinib | 4.9 | 73.3 | 4.9 | 4.9 | 3.3 | 7.3 | 1.5 | 3.3 | 25 |
|  | Vinorelbine | 13.2 | 234.9 | 13.1 | 13.3 | 8.7 | 20.2 | 2.4 | 8.6 | 22 |
|  | Melphalan | 5.5 | 71.9 | 5.4 | 5.5 | 3.6 | 8.4 | 1.6 | 3.5 | 21 |
|  | Bortezomib | 3.6 | 35.5 | 3.6 | 3.6 | 2.3 | 5.7 | 1.2 | 2.3 | 20 |
|  | Trastuzumab | 2.0 | 9.0 | 2.0 | 2.0 | 1.3 | 3.2 | 0.7 | 1.3 | 19 |
|  | Sunitinib | 5.6 | 63.2 | 5.6 | 5.6 | 3.5 | 8.9 | 1.5 | 3.5 | 18 |
|  | Azacitidine | 2.3 | 5.1 | 2.3 | 2.3 | 1.2 | 4.4 | 0.6 | 1.2 | 9 |
|  | Epirubicin | 2.8 | 8.7 | 2.8 | 2.8 | 1.5 | 5.4 | 0.8 | 1.5 | 9 |
|  | Cabazitaxel | 15.2 | 78.9 | 15.1 | 15.2 | 7.2 | 32.0 | 1.9 | 7.2 | 7 |
|  | Gefitinib | 3.2 | 5.5 | 3.2 | 3.2 | 1.3 | 7.7 | 0.7 | 1.3 | 5 |
|  | Eribulin | 4.9 | 8.7 | 4.9 | 4.9 | 1.8 | 13.0 | 0.9 | 1.8 | 4 |
|  | Decitabine | 4.1 | 4.4 | 4.1 | 4.2 | 1.3 | 12.9 | 0.7 | 1.3 | 3 |
|  | Lanadelumab | 4.2 | 4.5 | 4.2 | 4.2 | 1.4 | 13.2 | 0.7 | 1.4 | 3 |
| Antipsoriatics | Calcipotriol | 6.0 | 20.2 | 6.0 | 6.0 | 2.7 | 13.4 | 1.2 | 2.7 | 6 |
| Antiseptics and disinfectants | Povidone-iodine | 6.5 | 13.5 | 6.5 | 6.5 | 2.4 | 17.4 | 1.0 | 2.4 | 4 |
| Antithrombotic agents | Epoprostenol | 2.9 | 6.9 | 2.9 | 2.9 | 1.4 | 6.1 | 0.7 | 1.4 | 7 |
|  | Warfarin | 184.5 | 557.4 | 184.2 | 193.4 | 70.9 | 528.0 | 2.8 | 67.5 | 4 |
|  | Edoxaban | 4.7 | 5.4 | 4.7 | 4.7 | 1.5 | 14.5 | 0.7 | 1.5 | 3 |
| Antivirals for systemic use | Ribavirin | 2.9 | 47.6 | 2.8 | 2.9 | 2.1 | 3.9 | 1.1 | 2.1 | 41 |
|  | Boceprevir | 6.8 | 33.8 | 6.8 | 6.8 | 3.4 | 13.6 | 1.4 | 3.4 | 8 |
| Beta blocking agents | Atenolol | 3.1 | 61.8 | 3.0 | 3.1 | 2.3 | 4.1 | 1.2 | 2.3 | 47 |
|  | Nebivolol | 2.6 | 9.6 | 2.6 | 2.6 | 1.5 | 4.8 | 0.8 | 1.5 | 11 |
| Bile and liver therapy | Ursodeoxycholic acid | 2.8 | 7.5 | 2.8 | 2.8 | 1.4 | 5.6 | 0.7 | 1.4 | 8 |
| Calcium channel blockers | Amlodipine | 3.7 | 354.8 | 3.6 | 3.7 | 3.2 | 4.3 | 1.6 | 3.1 | 191 |
|  | Felodipine | 8.4 | 76.9 | 8.3 | 8.4 | 4.9 | 14.5 | 1.8 | 4.8 | 13 |
| Calcium homeostasis | Calcitonin | 9.6 | 22.7 | 9.5 | 9.6 | 3.6 | 25.6 | 1.2 | 3.6 | 4 |
| Cardiac therapy | Nicorandil | 4.4 | 4.8 | 4.4 | 4.4 | 1.4 | 13.6 | 0.7 | 1.4 | 3 |
| Corticosteroids for systemic use | Prednisone | 2.3 | 91.8 | 2.3 | 2.3 | 1.9 | 2.8 | 1.0 | 1.9 | 131 |
|  | Dexamethasone | 2.5 | 83.4 | 2.5 | 2.5 | 2.0 | 3.1 | 1.1 | 2.0 | 97 |
|  | Methylprednisolone | 2.1 | 17.1 | 2.1 | 2.1 | 1.5 | 2.9 | 0.7 | 1.4 | 33 |
|  | Hydrocortisone | 2.7 | 24.7 | 2.7 | 2.7 | 1.8 | 4.0 | 1.0 | 1.8 | 25 |
|  | Cortisone | 4.3 | 14.4 | 4.3 | 4.3 | 2.0 | 9.0 | 1.0 | 2.0 | 7 |
|  | Hydrocortisone succinate | 6.8 | 9.5 | 6.8 | 6.8 | 2.2 | 21.0 | 0.9 | 2.2 | 3 |
| Corticosteroids, dermatological preparations | Desoximetasone | 5.5 | 21.3 | 5.5 | 5.5 | 2.6 | 11.5 | 1.2 | 2.6 | 7 |
| Cough and cold preparations | Codeine | 2.9 | 48.0 | 2.9 | 2.9 | 2.1 | 4.0 | 1.1 | 2.1 | 40 |
| Diuretics | Indapamide | 6.7 | 85.8 | 6.7 | 6.7 | 4.3 | 10.5 | 1.7 | 4.2 | 19 |
|  | Triamterene | 4.7 | 39.4 | 4.6 | 4.7 | 2.8 | 7.8 | 1.3 | 2.8 | 15 |
|  | Bendroflumethiazide | 6.0 | 40.4 | 5.9 | 6.0 | 3.3 | 10.8 | 1.4 | 3.3 | 11 |
|  | Xipamide | 23.2 | 85.1 | 23.2 | 23.3 | 9.7 | 56.2 | 1.9 | 9.6 | 5 |
| Drugs for acid related disorders | Rebamipide | 4.5 | 7.7 | 4.5 | 4.5 | 1.7 | 12.1 | 0.8 | 1.7 | 4 |
| Drugs for functional gastrointestinal disorders | Mebeverine | 15.8 | 55.1 | 15.7 | 15.8 | 6.6 | 38.1 | 6.5 | 6.5 | 5 |
| Drugs for obstructive airway diseases | Terbutaline | 5.3 | 10.1 | 5.3 | 5.4 | 2.0 | 14.3 | 0.9 | 2.0 | 4 |
| Drugs for treatment of bone diseases | Zoledronic acid | 30.9 | 8914.7 | 27.6 | 31.1 | 27.8 | 34.8 | 4.3 | 24.7 | 347 |
|  | Alendronic acid | 33.5 | 7260.7 | 30.9 | 33.7 | 29.7 | 38.4 | 4.4 | 27.2 | 251 |
|  | Denosumab | 4.8 | 462.8 | 4.6 | 4.8 | 4.1 | 5.7 | 1.9 | 4.0 | 162 |
|  | Pamidronic acid | 132.6 | 11915.4 | 128.7 | 137.0 | 111.4 | 168.6 | 5.7 | 104.6 | 95 |
|  | Ibandronate | 9.9 | 378.7 | 9.8 | 10.0 | 7.5 | 13.2 | 2.5 | 7.4 | 49 |
|  | Risedronic acid | 18.9 | 585.4 | 18.7 | 19.0 | 13.6 | 26.4 | 3.0 | 13.4 | 36 |
| Drugs used in diabetes | Gliclazide | 4.6 | 44.3 | 4.6 | 4.6 | 2.9 | 7.4 | 1.4 | 2.8 | 17 |
|  | Glyburide | 2.3 | 6.7 | 2.3 | 2.3 | 1.3 | 4.1 | 0.7 | 1.3 | 11 |
| Endocrine therapy | Letrozole | 5.3 | 159.6 | 5.2 | 5.3 | 4.0 | 7.0 | 1.8 | 4.0 | 48 |
|  | Tamoxifen | 8.3 | 157.3 | 8.2 | 8.3 | 5.6 | 12.2 | 2.1 | 5.6 | 26 |
|  | Anastrozole | 5.2 | 79.0 | 5.1 | 5.2 | 3.5 | 7.6 | 1.6 | 3.5 | 25 |
|  | Fulvestrant | 4.8 | 64.5 | 4.7 | 4.8 | 3.2 | 7.2 | 1.5 | 3.2 | 23 |
|  | Exemestane | 4.9 | 42.4 | 4.9 | 4.9 | 2.9 | 8.1 | 1.4 | 2.9 | 15 |
|  | Bicalutamide | 4.2 | 16.3 | 4.2 | 4.2 | 2.1 | 8.4 | 1.0 | 2.1 | 8 |
|  | Goserelin | 3.4 | 9.5 | 3.4 | 3.4 | 1.6 | 7.1 | 0.8 | 1.6 | 7 |
|  | Toremifene | 47.4 | 138.0 | 47.3 | 47.9 | 17.9 | 128.5 | 2.1 | 17.6 | 4 |
| Immune sera and immunoglobulins | Human immunoglobulin G | 2.1 | 8.5 | 2.1 | 2.1 | 1.3 | 3.4 | 0.7 | 1.3 | 17 |
| Immunostimulants | Peginterferon alfa-2a | 3.2 | 41.4 | 3.2 | 3.2 | 2.2 | 4.6 | 1.2 | 2.2 | 29 |
|  | Filgrastim | 2.0 | 4.4 | 2.0 | 2.1 | 1.1 | 3.8 | 0.6 | 1.1 | 10 |
|  | Peginterferon alfa-2b | 3.3 | 13.8 | 3.3 | 3.3 | 1.8 | 6.2 | 0.9 | 1.8 | 10 |
| Immunosuppressants | Etanercept | 2.2 | 127.7 | 2.1 | 2.2 | 1.9 | 2.5 | 0.9 | 1.8 | 218 |
|  | Methotrexate | 2.3 | 100.4 | 2.2 | 2.3 | 1.9 | 2.7 | 1.0 | 1.9 | 151 |
|  | Cyclophosphamide | 2.6 | 59.8 | 2.5 | 2.6 | 2.0 | 3.3 | 1.0 | 2.0 | 66 |
|  | Infliximab | 2.2 | 35.8 | 2.2 | 2.2 | 1.7 | 2.9 | 0.9 | 1.7 | 57 |
|  | Abatacept | 2.5 | 38.7 | 2.5 | 2.5 | 1.9 | 3.4 | 1.0 | 1.9 | 45 |
|  | Cyclosporine | 2.5 | 32.2 | 2.5 | 2.5 | 1.8 | 3.4 | 0.9 | 1.8 | 39 |
|  | Leflunomide | 2.9 | 41.4 | 2.9 | 2.9 | 2.1 | 4.1 | 1.1 | 2.1 | 35 |
|  | Thalidomide | 5.1 | 95.8 | 5.0 | 5.1 | 3.5 | 7.2 | 1.6 | 3.5 | 31 |
|  | Golimumab | 3.0 | 35.5 | 3.0 | 3.0 | 2.1 | 4.4 | 1.1 | 2.1 | 28 |
|  | Everolimus | 2.8 | 27.4 | 2.8 | 2.8 | 1.9 | 4.2 | 1.0 | 1.9 | 25 |
|  | Sirolimus | 4.7 | 37.3 | 4.7 | 4.7 | 2.8 | 8.0 | 1.3 | 2.8 | 14 |
|  | Baricitinib | 4.5 | 15.5 | 4.5 | 4.5 | 2.1 | 9.4 | 1.0 | 2.1 | 7 |
|  | Antithymocyte immunoglobulin | 4.1 | 4.3 | 4.1 | 4.1 | 1.3 | 12.8 | 0.7 | 1.3 | 3 |
| Lipid modifying agents | Simvastatin | 2.2 | 46.4 | 2.2 | 2.2 | 1.8 | 2.8 | 0.9 | 1.7 | 73 |
|  | Ezetimibe | 2.1 | 12.2 | 2.1 | 2.1 | 1.4 | 3.1 | 0.7 | 1.4 | 24 |
|  | Omega-3-acid ethyl esters | 3.3 | 7.3 | 3.3 | 3.3 | 1.5 | 7.3 | 0.8 | 1.5 | 6 |
| Mineral supplements | Calcium | 2.1 | 31.1 | 2.1 | 2.1 | 1.6 | 2.7 | 0.8 | 1.6 | 56 |
|  | Ferrous | 3.0 | 20.2 | 3.0 | 3.0 | 1.8 | 4.8 | 1.0 | 1.8 | 17 |
|  | Potassium iodide | 6.7 | 9.3 | 6.7 | 6.7 | 2.2 | 20.8 | 0.9 | 2.1 | 3 |
|  | Potassium nitrate | 30.1 | 57.8 | 30.1 | 30.3 | 9.7 | 94.5 | 1.6 | 9.7 | 3 |
| Muscle relaxants | Carisoprodol | 2.9 | 6.8 | 2.9 | 2.9 | 1.4 | 6.1 | 0.7 | 1.4 | 7 |
|  | Metaxalone | 3.9 | 5.9 | 3.9 | 3.9 | 1.5 | 10.4 | 0.7 | 1.5 | 4 |
| Not classified | Lanolin | 81.4 | 242.0 | 81.3 | 83.0 | 30.8 | 223.7 | 2.4 | 30.2 | 4 |
| Other cardiac preparations | Coenzyme Q10 | 4.8 | 11.4 | 4.8 | 4.8 | 2.0 | 11.5 | 0.9 | 2.0 | 5 |
| Other nervous system drugs | Varenicline | 2.1 | 21.5 | 2.1 | 2.1 | 1.6 | 2.9 | 0.8 | 1.5 | 38 |
|  | Nicotine | 2.3 | 20.5 | 2.3 | 2.3 | 1.6 | 3.4 | 0.8 | 1.6 | 29 |
| Peripheral vasodilators | Pentoxifylline | 4.9 | 5.7 | 4.9 | 4.9 | 1.6 | 15.1 | 0.7 | 1.6 | 3 |
| Psychoanaleptics | Amitriptyline | 2.6 | 28.1 | 2.6 | 2.6 | 1.8 | 3.7 | 0.9 | 1.8 | 31 |
|  | Doxepin | 4.1 | 15.7 | 4.1 | 4.1 | 2.0 | 8.2 | 1.0 | 2.1 | 8 |
|  | Lofepramine | 192.6 | 770.0 | 192.3 | 202.4 | 82.3 | 497.4 | 3.1 | 8.2 | 5 |
| Psycholeptics | Lorazepam | 2.9 | 66.9 | 2.8 | 2.9 | 2.2 | 3.7 | 1.2 | 2.2 | 58 |
|  | Haloperidol | 3.1 | 25.2 | 3.1 | 3.1 | 2.0 | 4.9 | 1.0 | 2.0 | 19 |
|  | Asenapine | 8.8 | 101.9 | 8.7 | 8.8 | 5.4 | 14.4 | 1.9 | 5.3 | 16 |
|  | Prochlorperazine | 2.9 | 15.2 | 2.9 | 2.9 | 1.7 | 4.9 | 0.9 | 1.7 | 14 |
| Sex hormones and modulators of the genital system | Conjugated estrogens | 2.2 | 15.9 | 2.2 | 2.2 | 1.5 | 3.2 | 0.8 | 1.5 | 27 |
|  | Medroxyprogesterone | 2.1 | 10.1 | 2.1 | 2.1 | 1.4 | 3.3 | 0.7 | 1.3 | 19 |
|  | Raloxifene | 3.5 | 11.8 | 3.5 | 3.5 | 1.7 | 7.0 | 0.9 | 1.7 | 8 |
|  | Desogestrel | 4.4 | 12.3 | 4.3 | 4.4 | 2.0 | 9.7 | 1.0 | 2.0 | 6 |
|  | Megestrol | 6.8 | 24.0 | 6.8 | 6.8 | 3.0 | 15.1 | 1.2 | 3.0 | 6 |
|  | Norgestimate | 3.5 | 8.5 | 3.5 | 3.5 | 1.6 | 7.9 | 0.8 | 1.6 | 6 |
| Stomatological preparations | Epinephrine | 2.9 | 26.1 | 2.9 | 2.9 | 1.9 | 4.5 | 1.0 | 1.9 | 22 |
|  | Chlorhexidine | 20.9 | 375.9 | 20.8 | 21.0 | 13.7 | 32.3 | 2.9 | 13.5 | 21 |
|  | Sodium fluoride | 15.5 | 94.2 | 15.5 | 15.6 | 7.8 | 31.2 | 2.0 | 7.7 | 8 |
|  | Stannous fluoride | 57.9 | 170.2 | 57.8 | 58.7 | 21.9 | 157.7 | 2.2 | 21.5 | 4 |
|  | Fluoride ion | 8.7 | 13.5 | 8.7 | 8.7 | 2.8 | 27.2 | 1.0 | 2.8 | 3 |
| Topical products for joint and muscular pain | Loxoprofen | 4.8 | 14.6 | 4.8 | 4.9 | 2.2 | 10.8 | 1.0 | 2.2 | 6 |
| Urologicals | Finasteride | 4.6 | 78.6 | 4.6 | 4.6 | 3.2 | 6.7 | 1.5 | 3.2 | 29 |
|  | Silodosin | 4.1 | 6.5 | 4.1 | 4.1 | 1.5 | 10.9 | 0.8 | 1.5 | 4 |
| Vaccines | Tozinameran | 10.4 | 25.2 | 10.4 | 10.4 | 3.9 | 27.8 | 1.3 | 3.9 | 4 |
| Vitamins | Cholecalciferol | 3.2 | 152.3 | 3.1 | 3.2 | 2.6 | 3.9 | 1.4 | 2.6 | 105 |
|  | Folic acid | 2.1 | 28.6 | 2.0 | 2.1 | 1.6 | 2.7 | 0.8 | 1.6 | 55 |
|  | Vitamin D | 2.5 | 36.3 | 2.5 | 2.5 | 1.9 | 3.4 | 1.0 | 1.8 | 42 |
|  | Ergocalciferol | 3.2 | 39.7 | 3.2 | 3.2 | 2.2 | 4.6 | 1.1 | 2.2 | 28 |
|  | Vitamin E | 3.5 | 14.0 | 3.5 | 3.6 | 1.8 | 6.8 | 1.0 | 1.8 | 9 |

ATC: Anatomical Therapeutic Chemical; PRR: Proportional reporting ratio; χ2: Chi square; RRR: Relative reporting ratio; ROR: Reporting odds ratio; IC: Information component; and EBGM: Empirical Bayes geometric mean.
